# Interplay of Darwinian Selection, Lamarckian Induction and Microvesicle Transfer on Drug Resistance in Cancer

**DOI:** 10.1038/s41598-019-45863-z

**Published:** 2019-06-27

**Authors:** Arturo Álvarez-Arenas, Ana Podolski-Renic, Juan Belmonte-Beitia, Milica Pesic, Gabriel F. Calvo

**Affiliations:** 1 Department of Mathematics & MOLAB-Mathematical Oncology Laboratory, University of Castilla-La Mancha, 13071 Ciudad Real, Spain; 2 Institute for Biological Research “Siniša Stanković”, University of Belgrade, Despota Stefana 142, 11060 Belgrade, Serbia

**Keywords:** Cancer models, Applied mathematics

## Abstract

Development of drug resistance in cancer has major implications for patients’ outcome. It is related to processes involved in the decrease of drug efficacy, which are strongly influenced by intratumor heterogeneity and changes in the microenvironment. Heterogeneity arises, to a large extent, from genetic mutations analogously to Darwinian evolution, when selection of tumor cells results from the adaptation to the microenvironment, but could also emerge as a consequence of epigenetic mutations driven by stochastic events. An important exogenous source of alterations is the action of chemotherapeutic agents, which not only affects the signalling pathways but also the interactions among cells. In this work we provide experimental evidence from *in vitro* assays and put forward a mathematical kinetic transport model to describe the dynamics displayed by a system of non-small-cell lung carcinoma cells (NCI-H460) which, depending on the effect of a chemotherapeutic agent (doxorubicin), exhibits a complex interplay between Darwinian selection, Lamarckian induction and the nonlocal transfer of extracellular microvesicles. The role played by all of these processes to multidrug resistance in cancer is elucidated and quantified.

## Introduction

Cancer is a multifactorial disease evolving from different genetic and epigenetic changes, while interactions among cancer cells and normal cells within the tumor microenvironment additionally contribute to its complexity. Understanding these processes is crucial for the development of efficient anticancer therapeutic strategies^1^. To elucidate the polymorphic progression displayed by tumors in their response to different treatments, a Darwinian-like evolutionary dynamics interpretation has often been invoked. Indeed, many solid evidences showed that cancers develop in a Darwinian manner via stochastic genetic and epigenetic changes^2–7^. According to this interpretation, tumor cells are subjected to a number of selective pressures which lead to the outgrowth of the fittest clones. Under the action of specific therapies, which operate as selective pressure agents, resistant progenies emerge from the tumor population.

When considering an heterogeneous tumor cell population in the light of the Darwinian evolutionary dynamics paradigm, selection can act both on cells carrying pre-existent random oncogenic mutations as well as those exhibiting *de novo* induced phenotypic variation. The phenotypic variability observed during the natural history of a tumor results from the inherent stochastic noise of gene expression^8,9^. The selected cells may subsequently expand contributing to the transformation towards a more severe pathology observed in clinical patients^10^. Under the the action of chemotherapeutic agents Darwinian selection gives rise to a, so-called, intrinsic resistance. But cancer cell clones extensively interact and modify each other giving rise to a cellular network that is continuously reprogramming itself^11–13^. Thus the understanding of how resistance to anticancer drugs occurs needs to be expanded along new pathways. As it was revealed by Pisco *et al*.^14^, besides Darwinian selection, another process may take place that contributes to drug resistance: Lamarckian induction. It was confirmed, via single-cell longitudinal monitoring, that in HL60 acute myeloid leukaemia cells, following administration of a chemotherapeutic agent (vincristine), a subpopulation of cells initially sensitive to the treatment acquired a transient-resistance. The resulting malignant resistant cells were not only selected by the treatment, through a Darwinian-like process, but were also promoted to become resistant through a Lamarckian-like process. In principle, both Darwinian and Lamarckian processes are expected to be present and hence contribute to drug resistance^15,16^.

To further complicate the picture, Lamarckian induction of resistance is not developed towards a single administered drug, but quite often to large panels of structurally and functionally unrelated drugs, thereby resulting in multidrug resistance (MDR). The MDR cell phenotype is mainly attributed to the overexpression of ATP Binding Cassette (ABC) membrane transporters such as P-glycoprotein (P-gp). These transporters are the first line of a cellular defence system extruding drugs and other substrates from the cytosol, in an ATP-dependent manner, and hence decreasing the intracellular drug accumulation. This fact often implies that cells overexpressing these membrane transporters reduce their proliferation rate at the cost of pumping out the cytotoxic agents. Overexpression of the drug efflux protein P-gp has been observed in many cancers including those originating from lung, breast, ovary and brain^17,18^.

Drug resistance in cancer is also strongly dependent on intercellular communication and the tumor microenvironment. Flow of information among cancer and normal cells involves both local and nonlocal interactions. Local or direct cell-to-cell communication encompasses mechanisms such as formation of gap junctions, tunneling nanotubes^19,20^ and even networks made of ultra-long cellular protrusions or microtubes^21^. These structures are known to be instrumental in the resilience of many cancers, not only to chemotherapy but also to radiotherapy. Nonlocal cell interactions, which comprise long-range cell signalling, delivery of soluble factors and exchange of extracellular vesicles, account for the active modulation of the tumor microenvironment^22^. Recent findings have confirmed the role of microvesicles (MVs) and exosomes in the intercellular communication among cancer cells^23–32^. Interestingly, by mimicking an infectious disease process, MVs shed by more aggressive donor cells may transfer different cellular components to less aggressive acceptor cells. The cargo of these MVs includes efflux membrane transporters, genetic information and transcription factors necessary for their production in the recipient cells, thus contributing to the spread of resistant phenotypes within the cancer cell population. One of the best characterised components that is directly transferred via MVs among subpopulations of cancer cells is P-gp^23,31,32^. In co-culture experiments by Bebawy *et al*.^23^ it was reported that a resistant acute lymphoblastic leukaemia cell line (VLB_100_), overexpressing the MDR1/P-gp gene, released and transferred MVs containing functional P-gp to a sensitive acute lymphoblastic leukaemia cell line (CCRF-CEM). In the course of about 4 hours, this process conferred increased resistance to the CCRF-CEM cells, which was subsequently confirmed by drug accumulation assays using two structurally and functionally unrelated P-gp fluorescent substrates, rhodamine 123 and daunorubicin, commonly employed in the assessment of the MDR phenotype. More recently, in ref.^31^ using non-small cell lung cancer cell lines NCI-H460 (sensitive) and NCI-H460/R (resistant) it was confirmed that extracellular MVs, shed by NCI-H460/R cells, were able to transfer their metabolic phenotype to the sensitive NCI-H460 cells, which resulted in an increase in their glycolytic capacity.

To further understand and quantify some of the above-mentioned processes that occur in drug resistance, a large number of mathematical approaches have been developed^33–40^. Most of the approaches presented in those works can be classified into mechanism-based models and data-driven prediction techniques. Mechanism-based models range from those consisting of sets of ordinary differential equations accounting for the cellular population dynamics along with the effects of chemotherapeutic agents^41,42^; partial differential equations that consider the spatial heterogeneity of the tumor cell density and the intratumoral drug concentration^15,43–45^; stochastic models which take into account the action of the tumor microenvironment in the adaptation of cell subpopulations and where the initial conditions do not completely determine the future configuration of the system^46–49^; agent-based methods which can incorporate drug resistance at multiple levels^50,51^; and molecular dynamics simulation which can capture the conformational changes and the fluctuation at the atomic scale of both the administered drugs and their targets^52,53^. Following a different rationale, data-driven prediction methods for identifying biomarkers involved in drug resistance have recently attracted considerable interest and comprise omics data-based node biomarker screening, static and dynamic network approaches for identifying edge and module biomarkers^54–57^.

**Figure 1:**
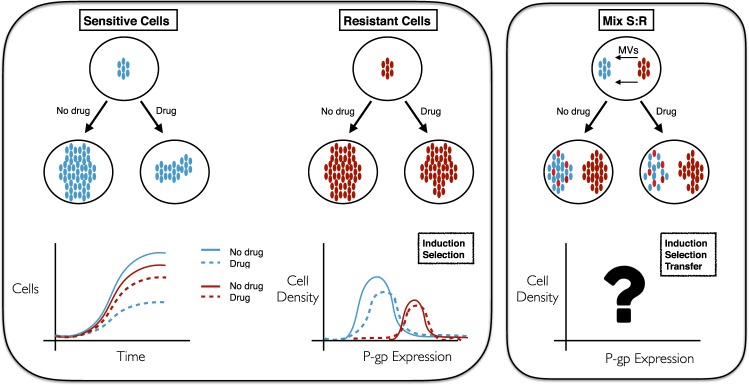
Possible scenarios displaying the response of sensitive and resistant cancer cell subpopulations to the absence/presence of a specific drug and intercellular communication. (Left panel) Selection and/or induction drive the cell number and P-gp expression kinetics when no communication exists between these two subpopulations. (Right panel) In addition to selection and/or induction, a transfer mechanism may arise when both subpopulations are in contact via an extracellular medium through which they can exchange microvesicles (MVs). Color code: blue and red for constitutively sensitive and resistant cells, respectively.

Herein, we put forward a mathematical kinetic transport-based framework which, from a unifying perspective, captures three different key processes involved in the development of drug resistance: Darwinian selection, Lamarckian induction and nonlocal transfer of extracellular MVs. One main goal of our approach is to quantify these three processes, and to elucidate in particular the contribution of the MVs carrying P-gp expression in the emergence of drug resistance. We analysed its importance both in the absence/presence of the chemotherapeutic agent doxorubicin (DOX) acting on sensitive human non-small cell lung carcinoma line NCI-H460 and its corresponding resistant cell line NCI-H460/R. Figure 1 summarizes the scope of the present study and depicts the mechanisms influencing P-gp expression in sensitive and resistant cancer cells.

To further address the raised questions, we performed a wide range of *in vitro* experiments using the NCI-H460 cell line (sensitive and resistant clones) and compared the results with *in silico* simulations of our mathematical model for its validation. Specifically, four experimental scenarios were considered:

1. Assessment of cell proliferation in real-time.
2. Analysis of changes in resistant phenotype of sensitive/resistant subpopulations using double staining.
3. Detection of P-gp transfer through both direct contact and indirect contact between sensitive and resistant cancer cells.
4. Duration of P-gp changes in the recipient cancer cells.

## Results

### DOX produces significant shifts in the P-gp expression levels of H460 cells only

**Figure 2:**
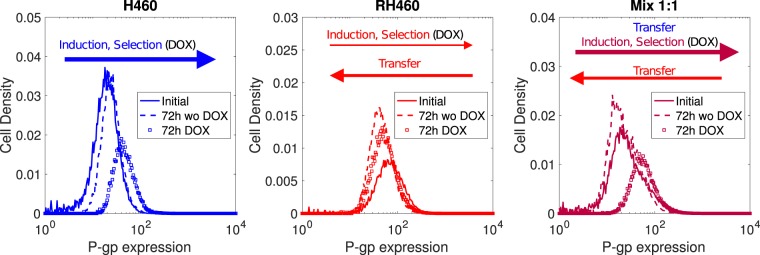
Experimental results for H460 (left), H460/R (middle) and mix 1:1 (right) cells. Arrows account for the processes that are responsible for the changes observed in P-gp expression. Notice that the areas under each curve, which represent the number of cells, are equal in all cases (all cell samples were of the same size $\simeq 2\times {10}^{4}$ in the flow cytometry analyses).

The distribution of P-gp in the different cell populations was assessed during four consecutive days to characterise their dynamics. Five initial proportions of sensitive (NCI-H460) and resistant (NCI-H460R) cells (S:R ratios equal to 1:0, 0:1, 1:1, 3:1, 7:1) were employed to analyse the changes in the P-gp expression both in the absence and presence of DOX (50 nM). Figure 2 shows how P-gp expression levels were modified in each cell population under various culture conditions and during a period of 72 h. For H460 cells, only in the presence of DOX there was a statistically significant shift towards higher P-gp expression levels (Fig. 2, left panel). For H460/R cells a slight shift towards lower P-gp expression levels appeared, although it was not statistically significant (Fig. 2, middle panel). For an initial 1:1 mixture of H460 and H460/R cells the kinetics was dramatically different in the absence/presence of DOX. Under DOX there was a statistically significant shift towards higher P-gp expression levels (Fig. 2, right panel). The corresponding *p*-values for all these experiments are displayed in Table S1 (Supplementary Information).

### Transport model captured the P-gp expression kinetics of all measured H460 and H460/R cell populations

**Figure 3:**
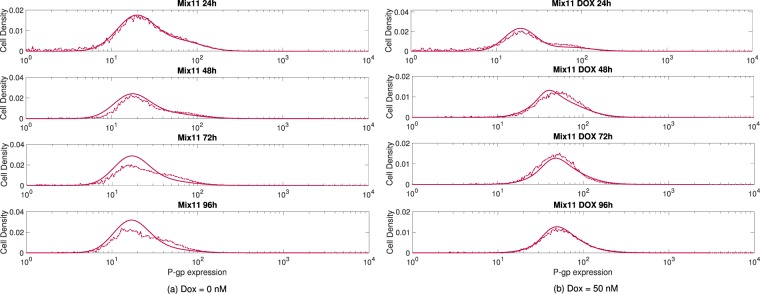
Evolution of H460 and H460/R cell mix 1:1 (**a**) without and (**b**) with DOX. Cells were seeded at *t* = 0 h and measured at subsequent intervals of 24 h. Dashed and solid lines represent the experimental results and the numerical simulations of our model, respectively.

Our mathematical model captured the experimentally observed cell growth kinetics of the different cell populations, both in the absence and in the presence of the drug DOX, and with various initial cell ratios (S:R ratios equal to 1:0, 0:1, 1:1, 3:1, 7:1). When assessing cell proliferation in real-time, a number of doses of DOX (0, 10, 50 and 100 nM) were used to quantify the effect over the total number of cells on an initial population of 4000 sensitive NCI-H460 cells via the xCELLigence Real Time Cell analyser. Our experimental results show that the higher the administered DOX doses were the slower was the cell growth (see Figs S4 and S5 in the Supplementary Information). This was most prominent for doses above 50 nM. These results allowed us to estimate the parameters entering into our model equations and specifically in the therapy function (see Methods and Supplementary Information), which accounts for the response to the administered chemotherapeutic agent with respect to the P-pg expression level. To minimize possible artefacts caused by nutrient depletion and release of metabolic products that eventually may become toxic, particularly when the cells reach confluence (which first occurred in the absence of DOX after 100 h, see Fig. S5), and may affect the antiproliferative activity of the drug^58,59^, all comparisons were made during the first 96 hours. Figure 3(a) evidences that our model was able to reproduce the experimental results without the drug, when the transfer of MVs among sensitive and resistant cells is the only process that could lead to alterations in the P-gp expression levels, but also in the presence of the drug (50 nM of DOX), where the processes of selection and induction become relevant, as depicted in Fig. 3(b). Supplementary Figs S6 to S13 further support the good agreement obtained between the model and the experiments for isolated sensitive and resistant cells as well as with different initial mixtures of these, both in the absence and in the presence of DOX. In the absence of the drug, there were no statistically significant changes in the P-gp expression levels whereas, under stress conditions, sensitive cells grown isolated or in mixtures showed highly significant changes in the observed P-gp level profiles (see Table S1 in the SI where *p*-values between initial and final conditions are collected). In contrast, resistant cells did not display any significant changes in their P-gp levels either in the absence or presence of the drug.

**Figure 4:**
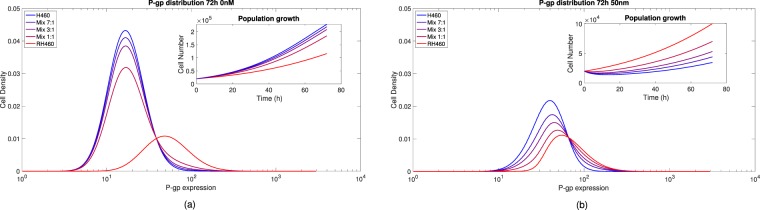
P-gp distribution changes after 72 h under (**a**) 0 nM and (**b**) 50 nM of DOX for initial sensitive, resistant and mixed cells (sensitive:resistant fractions 1:1, 3:1 and 7:1). Insets: Cell number versus time for initial populations of sensitive, resistant and mixed cells with 0 and 50 nM of DOX.

We used our model to analyse the role of initial conditions for the seeded cell populations on their subsequent P-gp expression dynamics. In Fig. 4 (see also Figs S6–S13 in the SI) a comparison of the P-gp expression with different ratios of sensitive and resistant cells is plotted together with the corresponding cell numbers during 72 h after seeding; the distinct colours encode the initial proportions (blue for sensitive only and light red for resistant only). Figure 4(a) shows that, in the absence of drug (0 nM of DOX), a small tail of cells having higher P-gp levels developed when the initial fraction of resistant cells was larger. Also, the cell number grows faster for sensitive cells and progressively decreases with the ratio of resistant cells (see inset of Fig. 4(a)). However, in the presence of drug (50 nM of DOX), see Fig. 4(b), for the very same initial proportions of Fig. 4(a), not only significant shifts in the P-gp expression profiles after 72 h were observed for initially sensitive cells and mixtures 1:1, 3:1 and 7:1 (see also Table S1 in the SI), but the corresponding cell numbers displayed noticeable differences. Except for resistant cells only, all mixtures evidenced a transient reduction in the cell numbers and, after a varying amount of hours depending on the initial ratios (see inset of Fig. 4(b)), the surviving tumor cells increased in all cases evidencing the emergence of subpopulations tolerant to DOX.

### The presence of DOX influences the rate of P-gp transfer to H460 cells

**Figure 5:**
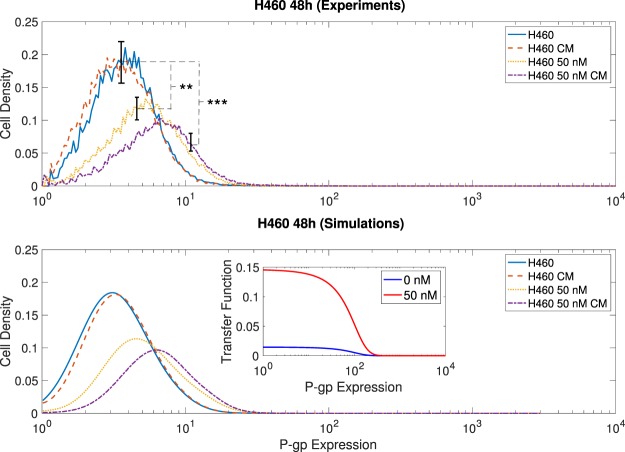
Detection of P-gp transfer during 48 h in different culture media with sensitive cells ($\simeq 2\times {10}^{4}$). Upper/lower rows represent the experimental results and the model predictions, with the corresponding calculated MV transfer function (inset). The culture media comprised only the H460 cells (blue solid curves), H460 cells grown in a conditioned medium (CM) exchanged from the H460/R cells to the H460 cells medium (red dashed curves), H460 cells grown in the presence of 50 nM of DOX (yellow dotted curves) and H460 cells grown both in the presence of 50 nM of DOX and CM (violet dashed-dotted curves). Asterisks in the upper row denote the *p*-values for pairwise comparisons: (**)*p* < 0.005 and (***)*p* < 0.0005.

Among all the processes implicated in the development of resistances, the one involving P-gp transfer via MVs was analysed independently. To this end, the P-gp expression of sensitive cells was measured both in standard and in conditioned media, the latter obtained from a culture of resistant cells. In previous works it has been demonstrated that MVs’ shedding constantly occurs among cancer cells^28^ and that MVs released by resistant cells carry P-gp molecules^31^. The upper row in Fig. 5 collects the results of both experimental conditions (with and without DOX) showing practically no difference in the absence of DOX. These differences were, however, statistically significant in the presence of the drug (at the significance level *α* = 0.05, see Table S2 in the SI), with an additional shift towards higher P-gp values when both DOX and the conditioned media from the resistant cells were combined. To test for reproducibility, three additional independent replicas of all these experiments were carried out. The results displayed very similar behaviour in all cases and identical conclusions were obtained in the statistical analyses (for details, see Fig. S14 in the SI).

The lower plots in Fig. 5 show that our model was also capable of reproducing the experiments of medium exchange in the presence and in the absence of DOX. One remarkable effect predicted by our model was the different MVs uptake rates displayed by sensitive cells depending on whether DOX was administered or not. The presence of MVs in the microenvironment appears to have a higher impact on the P-gp level of sensitive cells under the action of DOX. In the inset of Fig. 5 the transfer functions used in the simulations to fit the experimental data are plotted. Notice that the transfer function exhibits a ten fold change in the presence of the drug with respect to its absence. To support this finding, similar statistical analyses applied to the previous data were performed on the calculated distribution curves. Without DOX, the P-gp expression curves showed no significant difference between normal and conditioned medium. However, in the presence of DOX, our analysis displayed a difference at the level of *α* = 0.05 (see Table S2 in the SI) which was statistically significant.

### Increases in the P-gp expression levels of H460 cells were reversible

To answer the question of whether the observed changes in the P-gp expression levels of the initially sensitive cells, due to transfer, were permanent (within the considered time duration of our experiments) or else reversible, we performed an additional experiment outlined below. This is an important aspect from a clinical perspective as it has a direct impact when designing personalized dosing strategies. Indeed, if previously sensitive cells to a certain drug acquire a transfer-mediated-resistance phenotype which is transient, this implies that the initial drug may be employed repeatedly on those same cells once their characteristic return period to that drug has been identified. This is also at the heart of combining several drugs; the tumor cells exhibit distinct time-varying responses to each one of them but, in principle, while the different chemo agents are administered there will be no need to permanently discard subsets of these and introduce new ones. If, in contrast, previously sensitive cells to a certain drug, after acquiring a resistant phenotype, do not return to a sensitive state once the drugs are removed, then subsets of the employed chemo agents will be compromised and new therapeutical approaches will have to be developed for that specific tumor^16^.

**Figure 6:**
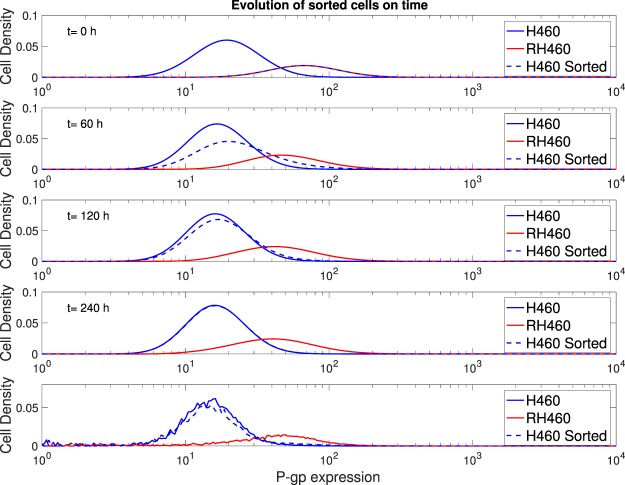
Duration of P-gp changes. The upper four rows correspond to the numerical simulations over a time period of 240 h, while the last row shows the experimental results (at *t* = 240 h).

Sensitive cells overexpressing P-gp were extracted and placed in a new fresh medium. Their resistance level was then monitored during 240 h. We observed that sensitive cells returned to their basal values of P-gp expression (see bottom row in Fig. 6). Therefore, the acquired transfer-mediated-resistance phenotype was reversible and did not involve genetic or epigenetic mutations. To quantify the duration of these changes, we fed our model with the same parameters used in previous simulations. The four upper rows in Fig. 6 depict snapshots at various times (*t* = 0, 60, 120, 240 h) of the expression levels corresponding to sensitive, resistant and sorted cells. At *t* = 0 h both resistant and sorted sensitive cells overexpressing P-gp show undistinguishable distributions. When placed in a new fresh medium (without any drug or MVs), the sorted sensitive cells overexpressing P-gp gradually recovered their basal distribution, which already at *t* = 60 h was manifestly shifted, being complete after ten days as in the experiments. Our model was able to mimic the observed changes without the need to modify the basal P-gp values; these were assumed to be time-independent during the entire examined period as any actual genetic mutation, possibly affecting these basal values, would be expected to take place at longer times (after many cell cycles).

### Variability in response to different treatment protocols is driven by initial percentage of resistant cells

To assess how the interplay of Lamarckian induction and nonlocal transfer of extracellular MVs both collectively and individually affect the progression of the tumor cell populations when subjected to different treatment schedules, we considered three protocols (Darwinian selection is always present). We used our model to simulate, during a time period of 240 h, how the total cell numbers changed upon DOX administration (see also Fig. S15) and compared the response with the growth in the absence of drug. The protocols that we examined were:

- Protocol 1: Drug during 0–120 h (no drug during 120–240 h), which corresponds to a single administration session.
- Protocol 2: Drug during 0–60 h and 180–240 h (no drug during 60–180 h), which corresponds to two administration sessions separated by one resting interval.
- Protocol 3: Drug during 0–24 h, 48–72 h, 96–120 h, 144–168 h and 192–216 h (no drug during the remaining 24 h intervals), which corresponds to five administration sessions alternated by four resting intervals.

Notice that the total time under drug pressure and drug absence is the same in the three protocols, 120 h and 120 h, respectively. In addition, each of the three protocols were simulated with three DOX concentrations of 10 nM, 50 nM and 100 nM (see Fig. S16 in SI).

**Figure 7:**
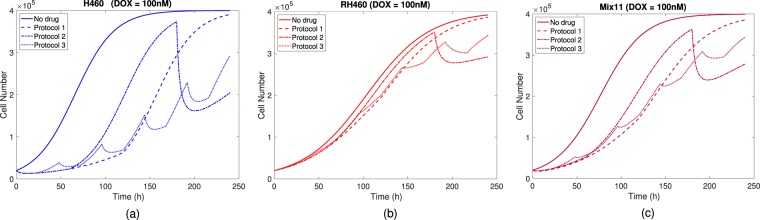
Treatment response of (**a**) H460, (**b**) RH460 and (**c**) Mix 1:1 cell groups to the three different protocols using a 100 nM drug concentration of DOX and a control group (no drug). The initial cell number was 2 × 10^4^ cells in all cases.

For initial sensitive cells subjected to 100 nM of DOX (see Fig. 7(a)), their response to the three protocols exhibits marked differences; protocol 1 results in a growth delay of about 120 h, protocol 2 shows the largest cell number change, whereas protocol 3 is the one giving rise to a slower growth on average with respect to the other two. In this scenario, induction is the most relevant process towards the emergence of resistance (MV transfer is essentially absent). Of the three protocols, number 2 is the one in which induction is smaller (see Fig. S17).

For initial resistant cells subjected to 100 nM of DOX (see Fig. 7(b)), the three protocols show much smaller differences with respect to the previous scenario as this subpopulation is expected to barely respond to treatment. In this case, the processes of induction and MV transfer do not play any relevant role whatsoever. The residual differences observed in the three protocols come from a small fraction of resistant cells having lower expression levels of P-gp which are be partially responsive to the drug.

When both sensitive and resistant cells are initially present and subjected to 100 nM of DOX (see Fig. 7(c)) both induction and MV transfer do contribute. Of the three protocols, number 2 is the one in which induction and MV transfer is larger (see Figs S17 and S18), hence more similar to the response shown by sensitive cells alone, while the responses to protocols 1 and 3 were more similar to that displayed by resistant cells alone.

Furthermore, when varying the doses and the protocols (see Figs S17 and S18), the largest observed differences occurred for sensitive cells alone and the smallest for resistant cells alone. Therefore, when a relatively large fraction of sensitive cells is present in the tumor, the response to therapy is more dependent on the specific protocol and the differences increase with dose concentration.

## Discussion

**Figure 8:**
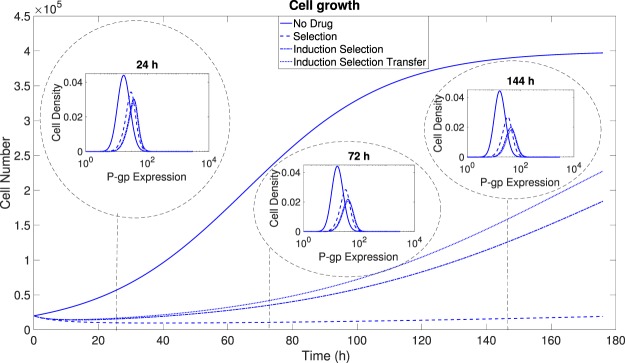
Portrait of the dynamics of P-gp expression and cell number under the action of Darwinian selection, Lamarckian induction and MV transfer on an initial population of sensitive cells with a continuous supply of MVs. The impact of these processes on P-gp expression and cell number can be quite dramatic in the presence of drugs (dotted, dashed-dotted and dashed curves). Darwinian selection yields the slowest dynamics whereas Lamarckian induction and MV transfer significantly accelerate the dynamics towards resistant populations.

We have presented both experimental evidence from *in vitro* assays and a mathematical framework to elucidate, from a unified perspective, three distinct processes implicated in the development of drug resistance: Darwinian selection, Lamarckian induction and nonlocal transfer of extracellular microvesicles (MVs). We have captured these three processes by means of an integro-differential transport model. Our mathematical model has been able not only to reproduce the obtained experimental results but also to quantify the relative relevance of these three processes. In particular, we have gone beyond previous studies by incorporating the additional process of P-gp transfer from resistant (acting as donor) to sensitive (acting as acceptor) cells mediated by the exchange of extracellular MVs. Our model has confirmed that this process, although relatively less important than selection and induction, has a cumulative effect during time and thus must be taken into account when describing the emergence of drug resistance in tumor cells lines known to release MVs to the extracellullar medium^23–32^. If Darwinian selection is the only process contributing to drug resistance its dynamics is expected to be dictated by both the initial fraction of resistant cells and the phenotypic diversity (e.g. the proliferation rate). In such a scenario the tumor progression may be considerably slow under the action of a drug if that initial fraction is very small and the proliferation rates of resistant and sensitive cells are quite disparate. However, even in the absence of any initial subpopulation over-expressing P-gp, the effect of non-genetic alterations can give rise to the development of resistance. In this scenario, Lamarckian induction becomes the most important process in the presence of drug, while the transfer of MVs contributes to the overall emergence of resistance becoming increasingly relevant when resistant cells exist within the tumor and shed extracellular MVs to the microenvironment. This implies that, in heterogeneous tumors, the role of extracellular MV transfer cannot be ignored and must be targeted by multidrug therapies. Figure 8 summarises all of these considerations.

One intriguing prediction of our model is that sensitive cells are more likely to accept extracellular MVs carrying P-gp under exogenous stress conditions. This preferential uptake of MVs by sensitive tumor cells opens new potential therapeutic routes to deal with MDR resistance in cancer. If this is an adaptive defensive mechanism by tumor cells against cytotoxic agents, this fact could be used to redesign treatments combining standard cytotoxic drugs with other agents encapsulated in liposomal nanoparticles which closely resemble MVs. Indeed, targeted delivery to mesenchymal stem cells via hybrid nanoparticles consisting of exosomes with liposomes enclosing large plasmids (a CRISPR-Cas 9 system) has recently been demonstrated^60^, thus the therapeutical avenue to exploit the preferential uptake by sensitive tumor cells of hybrid nanoparticles containing P-gp, acting as *Trojan horses*, seems to be within reach, although further experiments are required to finally confirm this hypothesis. Moreover, previous works have already remarked the importance of the microenvironment on transfer processes among cancer cells^25^, hence in cannot be discarded that our results, which have been validated *in vitro* could be even more relevant *in vivo*.

In addition, both our experimental and simulation results have revealed that the duration of the changes in the P-gp expression levels in those non-small-cell lung carcinoma cells (NCI-H460) that are initially sensitive to doxorubicin (DOX) may be transient and thus reversible. Originally sensitive cells which acquired a relatively high expression of P-gp in the presence of resistant cells, revert to their basal level of P-gp expression either when they are subsequently separated from resistant cells or released from a conditioned medium (enriched with extracellular MVs) in which resistant cells were cultured. However, it should be pointed out that this reversibility is not ubiquitous; we have recently found^16^ that in the human glioma cell line U251, under the administration of the alkylating agent temozolomide (TMZ), which is the chemotherapeutic standard of care for glioblastoma patients, the epigenetic changes experienced by those glioma cells initially sensitive to TMZ remain when the selective pressure exerted by TMZ is removed.

Moreover, our simulations with different therapeutic protocols highlight not only the importance of the proper administration timing, but also the high variability in response as a consequence of Lamarkian induction and MV transfer, which could range from tumor control to treatment failure. Furthermore, when dealing with highly resistant tumors our simulations suggest that quite disparate protocols may result in very similar outcomes, suggesting that treatment could be chosen according to the patient’s needs to reduce secondary effects and possible complications. Drug administration is driven by drug resistance and, as we have shown, processes such as MV transfer could be reversible in time in a number of tumor types thus providing avenues for better control without resorting to new chemotherapeutic agents. Dose spacing and strategic treatment interruption might be helpful to avoid the development of resistances in some tumors, particularly if these are mediated by reversible MV transfer.

### Concluding remarks

In conclusion, the mathematical framework put forward in the present work constitutes a first step to provide an unified approach encompassing Darwinian selection, Lamarckian induction and the transfer of extracellular MVs. We have quantified different experimental configurations characteristic of heterogeneous tumors to reveal how they respond to the action of cytotoxic drugs and how resistance emerges. While DOX was the administered drug in the cultures with the non small cell lung carcinoma cell line NCI-H460, more generally, all cell lines overexpressing P-gp will show a similar response not only to DOX, but also to other drugs that are P-gp substrates (e.g., paclitaxel). All of our experimental findings support the predictions of our mathematical kinetic transport model. This opens the path to many further explorations on different cancer types (e.g., breast, pancreatic, glioma) where not only P-gp but other cell membrane transporters could be transferred among cells within the tumor microenvironment. Our framework can also be extended to novel forms of resistances that may arise in other therapeutic modalities such as for instance under immunotherapy administration. In future works we plan to exploit our model to investigate a number of optimal therapeutic strategies to control the development of drug resistance in specific cancer types.

## Methods

### Cell lines

To study the processes involved in development of drug resistance in a well-defined scenario, we considered a number of *in vitro* assays under various culture conditions of sensitive human non-small cell lung carcinoma line NCI-H460 and its corresponding resistant cell line NCI-H460/R. NCI-H460 was purchased from the American Type Culture Collection, Rockville, MD. Exposure of NCI-H460 cells during three months to gradually increasing concentrations of the chemotherapeutic drug doxorubicin (DOX) resulted in the establishment of a new cell line (NCI-H460/R) resistant to DOX and other structurally and functionally unrelated drugs^61^. Therefore, our system comprises cancer cell lines that are either sensitive or resistant to DOX (the color code that we will use henceforth in the figures is blue and red for constitutively sensitive and resistant cells, respectively). NCI-H460 and NCI-H460/R cancer cell lines were maintained in a RPMI 1640 medium supplemented with 10% fetal bovine serum (FBS), 2 mM L-glutamine, and 10,000 U/ml penicilin, 10 mg/ml streptomycin, 25 *μ*g/ml amphotericin B solutions. Both cell lines were sub-cultured at 72 h intervals using 0.25% trypsin/EDTA and seeded into a fresh medium at the following densities: 8.0 × 10^3^ cells/cm^2^ for NCI-H460 and 1.6 × 10^4^ cells/cm^2^ for NCI-H460/R. The chemicals that were used: RPMI 1640 medium, FBS, antibiotic-antimycotic solution, L-glutamine and trypsin/EDTA were purchased from Bioind, Beit Haemek, Israel. Rhodamine 123 (Rho123) was obtained from Sigma-Aldrich Chemie Gmbh, Germany. FITC-conjugated anti-P-gp antibody was purchased from BD Biosciences, United Kingdom, while CellTrace™ Violet reagent was obtained from Molecular Probes, Life Technologies, Carlsbad, CA.

### Assessment of cell proliferation in real-time

Continuous cell proliferation of NCI-H460 and NCI-H460/R cancer cells untreated and treated with DOX after 24 h from initial seeding (10, 50, 100, 500 and 1000 nM) was analysed using the xCELLigence Real Time Cell analyser (ACEA Biosciences Inc., USA) which facilitates label free real-time cell analysis by measuring impedance-based signals across a series of gold electrodes. Using E-plates, 50 *μ*L of complete medium, RPMI 1640 was added to each well and the electrodes were allowed to stabilise for 30 min. The plates were then moved into the xCELLigence Real Time Cell analyser to set a base line without cells. The cells were then seeded on an E-plate at the following densities: 4.0 × 10^3^ and 8.0 × 10^3^ cells in a 200 *μ*l medium per well. Cells on the electrodes were monitored by reading and recording the cell impedance every 30 min through 338 sweeps.

### Analysis of changes in resistant phenotype in sensitive/resistant subpopulations using double staining

Flow-cytometry was employed to analyse the P-gp expression level as a measure of the resistant phenotype in mixes of cancer cells comprised of NCI-H460 cells labelled with vital dye, and unlabelled NCI-H460/R. Sensitive NCI-H460 cells were labelled with 5 *μ*M of the CellTrace™ Violet reagent for 20 min at 37 °C in 5% CO_2_. The cells were then washed three times with PBS and covered with fresh complete RPMI 1640 medium. Either CellTrace™ Violet labelled NCI-H460 or unlabelled NCI-H460/R, or a carefully homogenised mixture of labelled NCI-H460 and unlabelled NCI-H460/R in ratios 1:1, 3:1 and 7:1 were seeded in 6-well plates. After staining with FITC-conjugated anti-P-gp antibody, untreated samples and samples treated with 50 nM DOX (immediately after seeding) were analysed at time points: 24 h, 48 h, 72 h and 96 h on FacsAria III flow-cytometer (BD Bioscienses, San Diego, USA). The fluorescence of CellTrace™ Violet was assessed on the fluorescence channel DAPI-A and FITC-conjugated anti-P-gp antibody was evaluated on fluorescence channel FITC-A. A minimum of 2.0 × 10^4^ events were assayed for each sample.

### Detection of P-gp transfer

Here we considered two experimental settings. In a first setting, the medium was exchanged between different samples. The samples that were used in the medium exchange experiments were: NCI-H460 cells untreated and treated with 50 nM DOX (immediately after seeding) as well as NCI-H460/R cells untreated and treated with 50 nM DOX (immediately after seeding). Always, the medium from NCI-H460/R cells was transferred to the NCI-H460 cells. Thus, the medium from NCI-H460 cells grown for 24 h was replaced by the medium from NCI-H460/R cells grown for 24 h without or in the presence of 50 nM DOX. Similarly, the medium from NCI-H460 cells grown for 24 h in the presence of 50 nM DOX was replaced by the medium from NCI-H460/R cells grown for 24 h without or in the presence of 50 nM DOX. In a second experimental setting, NCI-H460 cells were treated immediately after seeding with 50 and 100 nM DOX and the P-gp expression was followed in untreated and treated cells at different time points. P-gp expression was assessed at time points 24 h, 48 h, 72 h, 144 h and in samples in which the medium was refreshed after 72 h and cells were left to grow additional 72 h (for a total of 144 h), as well as in samples in which the cells were transferred to a new well with fresh medium after 144 h and then left to grow additional 96 h (for a total of 240 h).

The cells were seeded at the beginning of all experiments in 6-well plates at 10^5^ cells in 1 ml medium. Cells were then collected by trypsinization, washed in cold PBS and directly immuno-stained with FITC-conjugated anti-P-gp antibody. After 90 min in dark at room temperature, the cells were pelleted by centrifugation, washed twice in cold PBS and then placed in it. The samples were kept on ice in the dark until the analysis on CyFlow Space Partec (Sysmex Partec GmbH, Germany). The fluorescence of FITC-conjugated anti-P-gp antibody was assessed on fluorescence channel 1 (FL1-H). A minimum of 10^4^ events were assayed for each sample and the obtained results were analysed using Partec FloMax software.

### Sorting of double stained cells: duration of P-gp changes

A carefully homogenized mixture of labelled NCI-H460 and unlabelled NCI-H460/R cells in ratio 1:1 was seeded in 75 cm^2^ flask at a density of 1.0 × 10^6^ cells in a 10 ml medium. After 72 h, the staining protocol with FITC-conjugated anti-P-gp antibody was performed and a sample of 2.0 × 10^5^ cells double stained with CellTrace™ Violet reagent and FITC-conjugated anti-P-gp antibody (approximately 2% of whole sample) was sorted in a sterile falcon tube with FBS on FacsAria III flow-cytometer (BD Bioscienses, San Diego, USA). The sorted cells were placed in the fresh medium and after 10 days of cultivation, these cells were characterized with respect to their Rho 123 accumulation and P-gp expression.

Rho123 accumulation was analysed by flow-cytometry utilizing the ability of Rho 123, which is a substrate for P-gp, to emit fluorescence. Studies were carried out on NCI-H460, NCI-H460/R and sorted cells initially seeded in 6-well plates at 10^5^ cells in a 1 ml medium. Cells were collected by trypsinization, resuspended in complete medium containing 5 *μ*M Rho123 and incubated at 37 °C in 5% CO_2_ for 30 min. At the end of the accumulation period, the cells were pelleted by centrifugation, washed twice with cold PBS and then placed in it. The samples were kept on ice in the dark until the analysis on FACSCalibur flow-cytometer (Becton Dickinson, Oxford, United Kingdom). The fluorescence of Rho123 was assessed on fluorescence channel 1 (FL1-H). A minimum of 10^4^ events were assayed for each sample and the obtained results were analysed using Cell Quest Pro Software (Becton Dickinson, Oxford, United Kingdom).

### Mathematical model

To capture in a unified framework the various experimental scenarios considered, we put forward a mathematical model based on the kinetic theory of a large number of active particles (tumor cells), with their individual biological state called *activity*^62^. Within this framework, the transfer of P-gp among the *in vitro* cells is regarded as a Markovian stochastic process. The mathematical model does not include spatial heterogeneity of the cell populations since the cell populations are well mixed and the spatial redistribution of MVs, which will be considered as the main mediators that drive the exchange of P-gp among cells, takes place at a much faster time scale than proliferation/apoptosis/mutations. However, our model does incorporate the genotypic and phenotypic heterogeneity encoded in the expression levels of P-gp. One key observation is that a subpopulation of cells may experience significant changes in its P-gp expression level. That is, initially it may display low values of P-gp, then temporarily show increased ones due to the presence of cytotoxic agents/other cell subpopulations, and subsequently reduce them in the absence of cytotoxic agents/other cell subpopulations. This suggests to introduce two P-gp related variables, one continuous and one discrete, with greatly disparate associated temporal variation. Cells having the same activity distribution will be assembled into functional subsystems distinguished by a discrete index, *i*, that will refer to their constitutive or intrinsic (genotypic) expression level of P-gp in the membrane in the absence of any external stimulus. Within each subpopulation, having the same discrete index *i*, a continuous variable, *x*, will account for the amount of P-gp in the cell membrane.

The state of each cell subpopulation is represented by the non-negative density function *u*_*i*_(*x*, *t*) which, at time *t*, has P-gp activity *x*, varying continuously on the interval [*x*_min_, *x*_max_], and a constitutive (i.e. genetically driven) P-gp level expression represented by the discrete index *i* = 1, 2, which stands for cell populations with low level (*i* = 1, i.e. sensitive) and high level (*i* = 2, i.e. resistant) expressions.

The two mentioned degrees of freedom reflect quite distinct time scales. On the one hand, the variable *x* can change in time due to both induction and microvesicle-mediated transfer of P-gp processes. The first requires the presence of a drug whereas in the second MVs are first secreted by donor cells and subsequently internalized into the membrane of acceptor cells. On the other hand, the index *i* labels the constitutive (or genetic) P-gp level expressed by the cells. This level may also change in time, but it requires mutations, and so it is much slower than the characteristic time of variation of *x*.

In the evolution equations for the density functions we will consider *long-range mean-field conservative interactions* among the cells. These mean-field conservative interactions will refer to two different processes. The first one will describe the interaction between cells and cytotoxic drugs present in the medium. When cells are not killed, they may express higher levels of P-gp due to the process of *Lamarckian Induction*. The second one will account for the exchange of a large number of MVs between pairs of cells. These two conservative interactions modify the P-gp expression levels of the interacting cells, but not their total number. In addition to these, our model takes into account proliferation/death interactions which do change the number of cells.

In a unified description of all these intervening processes, our mathematical model consist of a system of hyperbolic partial integro-differential equations that comprises both conservative and non-conservative parts, and concisely reads as

1 $$\frac{\partial {u}_{i}}{\partial t}+{\mathscr{C}}(x,t;{\bf{u}})={\mathscr{N}}{\mathscr{C}}(x,t;{\bf{u}}),$$

where ${\mathscr{C}}[x,t;{\bf{u}}]$ denotes the conservative part, depending on *x*, *t* and the density functions **u** = {*u*_1_, *u*_2_}. This part, which in general is nonlinear in **u**, describes the phenotypic changes in the expression level of P-gp which preserve the overall cell population. The non-conservative part ${\mathscr{N}}{\mathscr{C}}[x,t;{\bf{u}}]$, which is both nonlinear and nonlocal in **u**, accounts for proliferation and death mechanisms (including the action of cytotoxic drugs), and thus in principle does not preserve the overall cell population. The details of how the conservative and non-conservative parts ${\mathscr{C}}(x,t;{\bf{u}})$ and ${\mathscr{N}}{\mathscr{C}}[x,t;{\bf{u}}]$, respectively, are constructed are provided in the SI.

Our resulting system of integro-differential kinetic transport equations that govern the two cell subpopulations is

2 $$\begin{array}{rcl}\frac{\partial {u}_{i}}{\partial t}+\frac{\partial }{\partial x}\,({v}_{i}(x,t;{\bf{u}}){u}_{i}(x,t)) & = & {\int }_{{x}_{{\rm{\min }}}}^{{x}_{{\rm{\max }}}}\,{{\mathscr{W}}}_{i}(x,x^{\prime} ,t;{\bf{u}}){u}_{i}(x^{\prime} ,t)dx^{\prime} \\  &  & -\,{g}_{i}(t;{\bf{u}}){u}_{i}(x,t)-{{\mathscr{T}}}_{i}(x,t){u}_{i}(x,t),\end{array}$$

together with the equation that describes the kinetics of the concentration of MVs

3 $$\frac{dM}{dt}={\int }_{{x}_{min}}^{{x}_{max}}\,({{\rm{\Gamma }}}_{1}(x;M(t)){u}_{1}(x,t)+{{\rm{\Gamma }}}_{2}(x;M(t)){u}_{2}(x,t))dx\mathrm{.}$$

On the left-hand-side of Eq. (2), ${v}_{i}={v}_{{T}_{i}}+{v}_{{I}_{i}}$, with ${v}_{{T}_{i}}$ and ${v}_{{I}_{i}}$ being the transfer and the induction velocities, respectively. On the right-hand-side of Eq. (2), ${{\mathscr{W}}}_{i}(x,x^{\prime} ,t;{\bf{u}})$ and ${g}_{i}(t;{\bf{u}})$ denote the proliferation kernel and a decay function. The kernel provides a measure of how the heterogeneity in the activity *x*′ of the parent cells influences their proliferation rate and the activity levels *x* displayed by the daughter cells. The last term on the right-hand-side of Eq. (2) describes the phenotypic selection effect exerted by the chemotherapeutic agent (e.g. DOX, paclitaxel, epirubicin, etoposide, vinblastine, etc) on the cell subpopulations with P-gp level *x*. Since a different cytotoxic response is expected according to *x*, which is related to the ability of the cells to efflux the drug, we make explicit this dependence in the *therapy function* ${{\mathscr{T}}}_{i}(x,t)$, which can also vary with time if the chemotherapeutic agent is (or a combination of drugs are) administered according to some given schedule. All the above mechanisms act extragenetically; they do not involve any mutations of the genes that regulate the expression level of P-gp within the time scales studied here (about 100 hours which corresponds to the duration of our *in vitro* experiments). However, additional coupling terms may be incorporated to model the genotypic changes $i\leftrightarrow j$ for longer time scales.

It should be stressed that in (2) the advection term and the right-hand side terms have an antagonistic effect. The advection term will favour the shift of the cell distribution *u*_*i*_(*x*, *t*) towards the other *u*_*i*′_(*x*, *t*) whereas the right-hand side terms will tend, on average, to maintain each *u*_*i*_(*x*, *t*) around its basal value (in the absence of therapy). If the velocities *v*_*i*_(*x*, *t*; **u**) are not sufficiently large then no significant displacements in the cell distribution *u*_*i*_(*x*, *t*) will be observable. In the specific case of *u*_1_(*x*, *t*), this amounts to saying that no emergence of drug resistance will occur in any fraction of this cell subpopulation. Also, notice that, with respect to the transfer process, the shift of the MV acceptor cells towards higher values of *x* is not instantaneous, but is driven by the buildup of *M*(*t*) through Eq. (3). We also assume that the MVs diffuse through the culture medium at a time scale much shorter than that of cell proliferation. The initial condition for *M*(*t*) is zero for a fresh medium, but can be nonzero for a *conditioned* culture medium. The numerical methods used to solve our model Eqs (2) and (3), together with the statistical and sensitivity analysis, are summarized in the SI.

## Supplementary information

Supplementary Information

## Data Availability

This article has no additional data.
