# Supplementary material for: Interplay of Darwinian Selection, Lamarckian Induction and Microvesicle Transfer on Drug Resistance in Cancer
Source: Sci Rep. 2019 Jun 27;9:9332. doi: 10.1038/s41598-019-45863-z (PMC6597577; doi:10.1038/s41598-019-45863-z)
Supplement: Supplementary file 1 — Supplementary Information [file 41598_2019_45863_MOESM1_ESM.pdf]

Supplementary Information

Interplay of Darwinian Selection, Lamarckian Induction and Microvesicle Transfer on Drug Resistance in Cancer

Arturo Álvarez-Arenas, Ana Podolski-Renic, Juan Belmonte-Beitia, Milica Pesic, and Gabriel F. Calvo

Additional figures and details of the methods are provided below.

Mathematical Model

In the evolution equations for the distribution functions we consider *long-range mean-field conservative interactions* [Bellomo *et al.*, 2008] between a *test* cell with activity x belonging to the i-th population, and a *field* cell with activity x' belonging to the j-th population. Since no explicit mechanical interactions among the cells are included, the mean-field conservative interaction will refer to the exchange of a large number of microvesicles (MVs) between pairs of cells and to the role of Lamarkian induction driven by the chemotherapy, that is, to an interaction which modifies the P-gp expression levels of the interacting cells, but not their number. In addition to these, our model will take into account proliferation/death interactions which do change the number of cells.

The state of each functional subsystem (cell subpopulation) is represented by the density function

$$u_i(x,t) : [x_{\min}, x_{\max}] \times [0,T] \to \mathbb{R}^+ \,, \tag{S1}$$

which, at time t, has P-gp activity x, varying continuously on the interval $[x_{\min}, x_{\max}]$, and a constitutive (i.e. genetically driven) P-gp level expression represented by the discrete index $i = 1, 2$, which stands for cell populations with low level ($i = 1$) and high level ($i = 2$) expressions. More specifically, x gives a measure of the amount of P-gp per surface area of the cell. Thus, $u_i(x,t)dx$ represents the number of cells of genotype i which, at time t, have a P-gp activity between x and $x + dx$. Figure S1 depicts the characteristic normalized (i.e. having equal areas) distributions for each constitutive level expression.

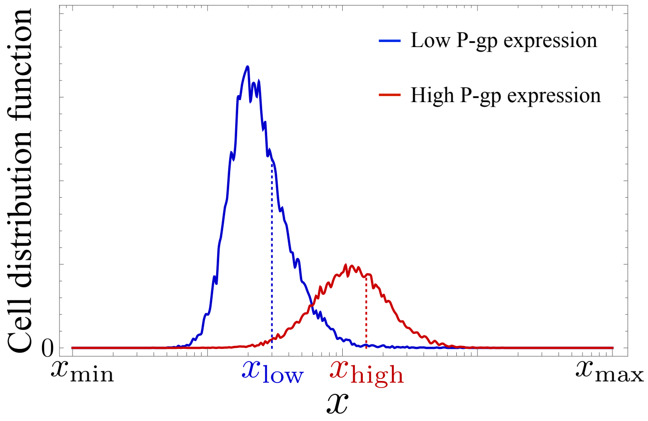

Figure S1: Density functions of the cell subpopulations with constitutively low and high expression levels of P-gp. Here x_{low} and x_{high} represent the corresponding mean values for each cell density. Also, the detection limits $x_{\min}$ and $x_{\max}$ in flow cytometry measuremens would correspond to $x_{\min} = 1$ and $x_{\max} = 10^4$. Notice that the horizontal axis is in a log scale.

The two mentioned degrees of freedom reflect quite distinct time scales. On the one hand, the variable x, that accounts for the P-gp expression, can change in time due to both induction and microvesicle-mediated transfer of P-gp processes. The first requires the presence of a drug whereas in the second MVs are first secreted by donor cells and subsequently internalized into the membrane of acceptor cells. On the other hand, the index i labels the constitutive (or genetic) P-gp level expressed by the cells. This level may also change in time, but it requires mutations, and so it is much slower than the characteristic time of variation of x.

The number of cells constitutively expressing low ($i = 1$) and high ($i = 2$) levels of P-gp at time t is given, respectively, by

$$n_i(t) = \int_{x_{\min}}^{x_{\max}} u_i(x,t)\, dx. \tag{S2}$$

In the evolution equations for the density functions we will consider *long-range mean-field conservative interactions* among the cells. These mean-field conservative interactions will refer to two different processes. The first one will describe the interaction between cells and cytotoxic drugs present in the medium. When cells are not killed, they may express higher levels of P-gp due to the process of *Lamarckian Induction.* The second one will account for the exchange of a large number of MVs between pairs of cells. These two conservative interactions modify the P-gp expression levels of the interacting cells, but not their number. In addition to these, our model will take into account proliferation/death interactions which do change the number of cells.

Our mathematical model consists of a system of hyperbolic partial differential equations that comprises both conservative and non-conservative parts, and concisely reads as

$$\frac{\partial u_i}{\partial t} + \mathcal{C}\,(x,t;\mathbf{u}) = \mathcal{NC}\,(x,t;\mathbf{u})\,, \tag{S3}$$

where $\mathcal{C}\,[x,t;\mathbf{u}]$ denotes the conservative part, depending on x, t and the density functions $\mathbf{u} = \{u_1, u_2\}$. This part, which in general is nonlinear in $\mathbf{u}$, describes the phenotypic changes in the expression level of P-gp which preserve the overall cell population. The non-conservative part $\mathcal{NC}\,[x,t;\mathbf{u}]$, which is both nonlinear and nonlocal in $\mathbf{u}$, accounts for proliferation and death mechanisms (including the action of cytotoxic drugs), and thus in principle does not preserve the overall cell population.

Cell-number conservative interactions

In the absence of the right hand-side, Eq. (S3) reduces to an advection equation. Upon integration of the advection term over x we get

$$\begin{aligned}\int_{x_{\min}}^{x_{\max}} \frac{\partial}{\partial x}(v_i(x,t)u_i(x,t))dx &= v_i(x_{\max},t;\cdot)u_i(x_{\max},t) \\ &- v_i(x_{\min},t;\cdot)u_i(x_{\min},t)\,. \end{aligned} \tag{S4}$$

In order for (S4) to vanish for all $t \geq 0$, we may impose Dirichlet boundary conditions $u_i(x_{\max},t) = u_i(x_{\min},t) = 0$ or else, $v_i(x_{\max},t;\cdot) = v_i(x_{\min},t;\cdot) = 0$. In either case, $n_i(t) = \int_{x_{\min}}^{x_{\max}} u_i(x,t)dx$ must remain constant in time if only the conservative part is present (there is only an internal redistribution of x within the populations).

To elucidate the form of Eq. (S3), let us first consider the conservative part. It will have the structure of an advection term

$$\mathcal{C}\,(x,t;\mathbf{u}) = \frac{\partial}{\partial x}(v_i(x,t;\mathbf{u})u_i(x,t))\,, \tag{S5}$$

where $v_i(x,t;\mathbf{u})$ is a velocity (it has the dimensions of activity or P-gp expression level over time) whose magnitude gives a measure of how prominent is the process of P-gp expression change in the cell subpopulations. This velocity encompasses the processes of *MV transfer* and *Lamarckian induction* and can be cast in the form

$$v_i = v_{T_i} + v_{I_i}, \tag{S6}$$

where v_{T_i} and v_{I_i} are the transfer and the induction velocities, respectively. Both velocity functions are continuous decreasing functions in $[x_{\min}, x_{\max}]$, with $v_{I_i} \geq 0 \;\; \forall x \in [x_{\min}, x_{\max}]$. The induction velocity v_{I_i} takes into account how the presence of the drug in the medium modifies (shifts) the expression levels of the P-gp without affecting to the total cell number. In the absence of the drug $v_{I_i} = 0$.

Let us now examine in more detail how the transfer velocity $v_{T_i}(x,t;\mathbf{u})$ depends on the cell subpopulations. This velocity describes the transfer of P-gp among cells. It gives rise to an x-dependent shift on the density functions. In contrast with v_{I_i}, notice that v_{T_i} can be positive or negative depending on whether the cells uptake or release MVs. Let $M(t)$ denote the number of MVs in the culture medium and define the functions $\Gamma_i\,(x; M(t))$ that account for the rate of uptake (negative) or shedding (positive) of MVs by the cell subpopulations depending on their x value. Notice that the $\Gamma_i\,(x; M(t))$ depend on $M(t)$ according to whether they represent adsorption or release processes of P-gp. The governing equation for $M(t)$ is

$$\frac{dM}{dt} = \int_{x_{\min}}^{x_{\max}} \left(\Gamma_1\,(x; M(t))\, u_1(x,t) + \Gamma_2\,(x; M(t))\, u_2(x,t)\right) dx. \tag{S7}$$

To properly choose Γ_1 and Γ_2 we refer to the following proposition.

Proposition 1. *Let $\mathcal{C}_{P\text{-}gp}(t)$ be defined as a measure of the total amount of P-gp in the system at time t, which can be formulated as follows*

$$\mathcal{C}_{P\text{-}gp}(t) = \alpha M(t) + \int_{x_{min}}^{x_{max}} x\,(u_1(x,t) + u_2(x,t))\, dx, \tag{S8}$$

where α is a constant that describes the amount of P-gp carried by the MVs. In the absence of the non-conservative part, if the rates Γ_i satisfy

$$\Gamma_i\,(x; M(t)) = -\frac{1}{\alpha} v_{T_i}(x,t;\mathbf{u}), \tag{S9}$$

then the total amount of P-gp remains constant in the system, i.e. $\frac{d\mathcal{C}_{P\text{-}gp}}{dt} = 0$.

Proof. Calculating the derivative in Eq.(S8) we get,

$$\frac{d\mathcal{C}_{\text{P-gp}}}{dt} = \alpha \frac{dM}{dt} + \int_{x_{\min}}^{x_{\max}} x \left(\frac{\partial u_1}{\partial t} + \frac{\partial u_2}{\partial t}\right) dx. \tag{S10}$$

We now make use of the following differential equation for the time variation of the MVs.

$$\frac{dM}{dt} = \int_{x_{\min}}^{x_{\max}} \left(\Gamma_1\,(x; M(t))\, u_1(x,t) + \Gamma_2\,(x; M(t))\, u_2(x,t)\right) dx, \tag{S11}$$

together with the advection equations (Eq.(S3) without the non-conservative part and $v_{I_i} = 0$) getting,

$$\frac{\partial u_i}{\partial t} + \frac{\partial}{\partial x}\left(v_{T_i}(x,t;\mathbf{u}) u_i(x,t)\right) = 0. \tag{S12}$$

Thus, Eq. (S10) transforms into

$$\begin{aligned}\frac{d\mathcal{C}_{\text{P-gp}}}{dt} &= \alpha\int_{x_{\min}}^{x_{\max}}\left(\Gamma_1\left(x;M(t)\right)u_1(x,t)+\Gamma_2\left(x;M(t)\right)u_2(x,t)\right)dx \\ &\quad -\int_{x_{\min}}^{x_{\max}} x\frac{\partial}{\partial x}\left(v_{T_1}(x,t;\mathbf{u})u_1(x,t)+v_{T_2}(x,t;\mathbf{u})u_2(x,t)\right)dx. \qquad \text{(S13)}\end{aligned}$$

Integration by parts of the right-hand-side and imposing the boundary conditions $u_i(x_{\max},t) = u_i(x_{\min},t) = 0$, yields

$$\begin{aligned}\frac{d\mathcal{C}_{\text{P-gp}}}{dt} &= \alpha\int_{x_{\min}}^{x_{\max}}\left(\Gamma_1\left(x;M(t)\right)u_1(x,t)+\Gamma_2\left(x;M(t)\right)u_2(x,t)\right)dx \\ &\quad +\int_{x_{\min}}^{x_{\max}}\left(v_{T_1}(x,t;\mathbf{u})u_1(x,t)+v_{T_2}(x,t;\mathbf{u})u_2(x,t)\right)dx. \qquad \text{(S14)}\end{aligned}$$

Now, regrouping members and using Eq. (S9) we conclude,

$$\frac{d\mathcal{C}_{\text{P-gp}}}{dt} = 0. \qquad \text{(S15)}$$

■

Equation (S9) shows that the velocities are proportional to the acceptor/release rates $\Gamma_i\left(x;M(t)\right)$ and depend on $u_i(x,t)$ via Eq. (S7). Without loss of generality, henceforth we will take $\alpha = 1$. Although the dependence of $v_{T_i}(x,t;\mathbf{u})$ on $\Gamma_i\left(x;M(t)\right)$ has been derived on the basis that the total amount of P-gp in the system remains constant, if we further assume that the production of P-gp takes place mainly during cell proliferation or otherwise at a time scale which is of the order of the cell cycle, we may approximately maintain the simple form embodied in (S9).

Let us specify in more detail the structure of the rate functions $\Gamma_i\left(x;M(t)\right)$ arising in our model. To this end, we identify threshold values $\tilde{x}_i$ such that if $x < \tilde{x}_i$ then $\Gamma_i\left(x;M(t)\right) < 0$ and if $x > \tilde{x}_i$ then $\Gamma_i\left(x;M(t)\right) > 0$. On the one hand, if cells have an expression level of P-gp below $\tilde{x}_i$ they will act as acceptors of MVs. For this to occur it is necessary that $M(t) > 0$, otherwise there cannot be any uptake of MVs. On the other hand, if cells have an expression level of P-gp above $\tilde{x}_i$ they will act as donors of MVs. We thus write

$$\Gamma_i\left(x;M(t)\right) = \begin{cases} \gamma_i(x)\chi_{M(t)} & x < \tilde{x}_i, \\ \gamma_i(x) & x > \tilde{x}_i, \end{cases} \qquad \text{(S16)}$$

where $\chi_{M(t)} = 1$ if $M(t) > 0$ and zero, otherwise. Notice that the functions $\gamma_i(x)$ are negative if $x < \tilde{x}_i$ and positive otherwise.

One important consequence of the above advection model, with respect to the transfer process, is that the shift of the MV acceptor cells towards higher values of x is not instantaneous, but is driven by the buildup of $M(t)$ through Eq. (S7). To conclude the analysis of the conservative part of the model, it has been implicitly assumed that the MVs diffuse through the culture medium at a time scale much shorter than that of cell proliferation. The initial condition for $M(t)$ is zero for a fresh medium, but can be nonzero for a *conditioned* culture medium.

Cell-number non-conservative interactions

We now turn to the non-conservative part $\mathcal{NC}\left[x,t;\mathbf{u}\right]$ in Eq. (S3). This part comprises all those contributions that imply a net change in each of the two cell subpopulations. The general structure is

$$\mathcal{NC}_i\left[x,t;\mathbf{u}\right] = \int_{x_{\min}}^{x_{\max}} \mathcal{W}_i(x,x',t;\mathbf{u})u_i(x',t)dx' - g_i(t;\mathbf{u})u_i(x,t) - \mathcal{T}_i(x,t)u_i(x,t). \quad \text{(S17)}$$

The first two terms of (S17) account for the net growth, via a proliferation kernel $\mathcal{W}_i(x,x',t;\mathbf{u})$ and a decay function $g_i(t;\mathbf{u})$. The kernel provides a measure of how the heterogeneity in the activity x' of the parent cells influences their proliferation rate and the activity levels x displayed by the daughter cells. The last term describes the phenotypic selection effect exerted by the chemotherapeutic agent (e.g. DOX, paclitaxel, epirubicin, etoposide, vinblastine, etc) on the cell subpopulations with P-gp level x. Since we expect a different cytotoxic response according to x, which is related to the ability of the cells to efflux the drug, we make explicit this dependence in the *therapy function* $\mathcal{T}_i(x,t)$, which can also vary with time if the chemotherapeutic agent is (or a combination of drugs are) administered according to some given schedule. All the above mechanisms act extragenetically; they do not involve any mutations of the genes that regulate the expression level of P-gp within the time scales studied here (about 100 hours which corresponds to the duration of our *in vitro* experiments). However, additional coupling terms may be incorporated to model the genotypic changes $i \leftrightarrow j$ for longer time scales.

Let us now detail the structure of the proliferation kernel $\mathcal{W}_i(x,x',t;\mathbf{u})$. It is given by

$$\mathcal{W}_i(x,x',t;\mathbf{u}) = 2\rho_i(\mathbf{u})f_i\left(x - x_i^{(\text{basal})} - \frac{x' - x_i^{(\text{basal})}}{\zeta_i}\right), \quad \text{(S18)}$$

where the factor $\rho_i(\mathbf{u})$ denotes the proliferation rate of subpopulation i and it may depend on the number of cells $\{n_1(t), n_2(t)\}$. The inclusion of a proliferation term depending on the number of cells has already been highlighted in previous works [Lavi *et al.*, 2013, Chisholm *et al.*, 2015] and takes into account the effect of population size and competition for resources within the culture medium.

The second important factor in the proliferation kernel is $f_i(\cdot)$, a conditional probability density function which accounts for the occurrence of a change in the expression level during mitosis. That is, from the parent cell with level x' to the daughter cells with level x. Also, the parameters $x_i^{(\text{basal})}$ and ζ_i denote the characteristic basal levels of P-gp for each cell subpopulation (with $x_1^{(\text{basal})} < x_2^{(\text{basal})}$ and the splitting factor of P-gp from the parent to the daughter cells ($\zeta_i \simeq 2$ for symmetric cell division), respectively. The function $f_i(\cdot)$ is a unimodal function (i.e., it has a single local extremum, in our case a maximum) and satisfies the normalization condition

$$\int_{x_{\min}}^{x_{\max}} f_i\left(x - x_i^{(\text{basal})} - \frac{x' - x_i^{(\text{basal})}}{\zeta_i}\right) dx = 1, \quad \text{(S19)}$$

reflecting the fact that, with probability one, the parent cell transitions from a state with expression level x' to a daughter cell with expression level $x \in [x_{\min}, x_{\max}]$. Note that, on the one hand, if the parent cell has a level $x' > x_i^{(\text{basal})}$ it will equally split (in the case of symmetric cell division) the P-gp content of its membrane around the

value $x = x_i^{(\text{basal})} + \frac{x' - x_i^{(\text{basal})}}{2}$ to its daughter cells, which is still larger than $x_i^{(\text{basal})}$ but smaller than x'. On the other hand, if the parent cell has a level $x' < x_i^{(\text{basal})}$, the daughter cells will have a value x smaller than $x_i^{(\text{basal})}$ although larger than x'. That is, the probability density function $f_i(\cdot)$ is assumed to display a stabilizing effect, meaning that in absence of exogenous agents and cell interactions, each cell subpopulation, after undergoing several cell cycles, will tend to be distributed around its basal value $x_i^{(\text{basal})}$, see Fig. S2. An important premise, which is implicit in the proliferation kernel, is that the dynamics of P-gp change occurs in a time scale of the order of the proliferation time τ_i, which is larger than the characteristic time scale during which MV redistribution takes place in the medium.

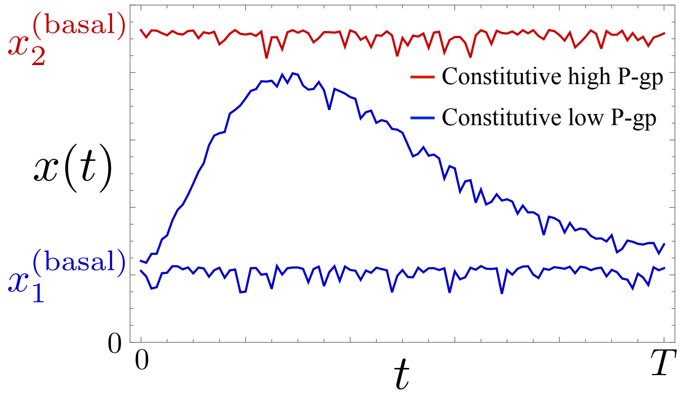

Figure S2: In the absence of exogenous agents and interactions, isolated subpopulations with constitutively low or high expression levels of P-gp will exhibit small fluctuations around their characteristic basal values $x_1^{(\text{basal})}$ and $x_2^{(\text{basal})}$, respectively. However, if an interaction between these cell subpopulations exists, those with constitutively low expression levels may transiently display higher values of P-gp for a time duration of the order of T. Such higher values will persist if the interaction between these subpopulations is sustained for sufficiently long times.

The decay function $g_i(t; \mathbf{u})$ is given by

$$g_i(t; \mathbf{u}) = \rho_i(\mathbf{u}) + \frac{1}{\tau_i^{(\text{death})}}, \tag{S20}$$

where the first term describes the losses when a cell with level x undergoes division into daughter cells with different P-gp level values (it is the inverse process embodied by the proliferation kernel), and the second term accounts for the intrinsic cell death with a characteristic death time $\tau_i^{(\text{death})}$.

In our numerical calculations, the factor $\rho_i(\mathbf{u})$ in the proliferation kernel will be further assumed to display a logistic form

$$\rho_i(\mathbf{u}) = \frac{1}{\tau_i}\left(1 - \frac{\sum_{j=1}^{2} n_j(t)}{n_{\max}}\right), \tag{S21}$$

where $n_j(t)$ is given by (S2) and τ_i denotes a characteristic proliferation time of subpopulation i, but other proliferation rates may be explored as well. The factor within parentheses is a saturation term that takes into account that the sum of the cell densities $n_j(t)$ cannot exceed the carrying capacity $n_{\max}$ of the culture medium.

To conclude the analysis of Eq. (S17), the therapy function $\mathcal{T}_i(x, t)$ is the one that brings out the key distinction between sensitive and resistant cells. Up to this point in

the modelling processes, we have not explicitly associated the labelling of sensitive and resistant cells to those of low and high expression levels of P-gp, respectively. Although it is indeed expected that cells expressing low levels of P-gp should correspond to the sensitive ones ($i = 1$), and those expressing high levels of P-gp should correspond to the resistant ones ($i = 2$), it is only by specifying the type of therapy that a suitable threshold between the two can be put down into quantitative form. In this way, when analysing our system, we may speak of cells which, although constitutively expressing low levels of P-gp, and thus expected to be sensitive to the chemotherapy (e.g. DOX), could evolve towards a (perhaps transient) state in which they would become resistant to therapy.

Kinetic transport equations

Combining the conservative and non-conservative terms, our resulting system of integro-differential equations that govern the two cell subpopulations are

$$\begin{aligned}\frac{\partial u_i}{\partial t} + \frac{\partial}{\partial x}(v_i(x,t;\mathbf{u})u_i(x,t)) \;=\; & \int_{x_{\min}}^{x_{\max}} \mathcal{W}_i(x,x',t;\mathbf{u})u_i(x',t)dx' - g_i(t;\mathbf{u})u_i(x,t) \\ & - \mathcal{T}_i(x,t)u_i(x,t), \end{aligned} \tag{S22}$$

together with the equation that describes the kinetics of the concentration of MVs

$$\frac{dM}{dt} = \int_{x_{min}}^{x_{max}} \left(\Gamma_1\left(x; M(t)\right) u_1(x,t) + \Gamma_2\left(x; M(t)\right) u_2(x,t)\right) dx. \tag{S23}$$

It should be stressed that in (S22) the advection term and the right-hand side terms have an antagonistic effect. The advection term will favour the shift of the cell distribution $u_i(x,t)$ towards the other $u_{i'}(x,t)$ whereas the right-hand side terms will tend, on average, to maintain each $u_i(x,t)$ around its basal value (in the absence of therapy). If the velocities $v_i(x,t;\mathbf{u})$ are not sufficiently large then no significant displacements in the cell distribution $u_i(x,t)$ will be observable. In the specific case of $u_1(x,t)$, this amounts to saying that no emergence of drug resistance will occur in any fraction of this cell subpopulation.

Numerical simulations

Our system of partial differential equations was first discretized, along the P-gp expression axis, by means of the method of lines. The resulting system of ordinary differential equations was then solved via a classic fourth-order Runge-Kutta method until convergence was attained (the temporal step used was $\Delta t = 10^{-3}$ h). Our numerical approach combined firstly an upwind scheme for the treatment of the P-gp expression variable and its derivatives within the conservative part. Secondly, the integrals of the nonlocal non-conservative parts were evaluated using Gauss-Legendre quadratures.

Statistical Analysis

The statistical analysis was carried out employing the Mann-Whitney U test (equivalent to the Wilcoxon rank sum test). This is a non-parametric test of the null hypothesis which states that it is equally likely that a randomly selected value from one sample will be less than or greater than a randomly selected value from a second sample. The Mann-Whitney U test does not require the assumption that the distributions are normal. In our case, we consider the following hypotheses:

- H_0: Both samples were selected from populations having identical distributions.
- H_1: Both samples were selected from populations having different distributions.

with a significance level $\alpha = 0.05$. The results are shown in Table S1.

Treatment	Sensitive	Resistant	Mix 1:1	Mix 3:1	Mix 7:1
No DOX	0.062	0.000***	0.026*	0.157	0.069
DOX	0.000***	0.000**	0.000***	0.000***	0.000***

Table S1: Statistical analysis (p-values) of the experimental results obtained for the changes in the P-gp expression levels after 72 h for sensitive/resistant/mixed cell cultures shown in Fig. 3 of the main text and Figs. S6-S13. Here, $p < 0.05$ (*), $^{**}p < 0.005$ (**), $^{***}p < 0.0005$ (***).

A similar statistical analysis was performed to detect the effect of extracellular microvesicles (MVs) on the P-gp level of sensitive cells under various culture conditions. Results are displayed in Table S2.

Treatment	H460	H460 CM	H460 DOX	H460 CM DOX
H460	-	0.600	0.000**	0.000***
H460 CM	-	-	0.000***	0.000***
H460 DOX	-	-	-	0.005*

Table S2: Statistical analysis (p-values) of the experimental results obtained to study the effect of extracellular MVs on the P-gp level of sensitive cells under various culture conditions after 48h. Here, a comparison of the P-gp levels of sensitive cells with and without changing the medium (CM) and in the presence/absence of drug (DOX) is made. The experimental results are shown in Fig. 5 of the main manuscript. Here, $p < 0.05$ (*), $^{**}p < 0.005$ (**), $^{***}p < 0.0005$ (***).

To further support this information, the Kolmogorov-Smirnov and the Cramer-von Mises tests were also used and we obtained identical conclusions in all cases.

Sensitivity Analysis

We addressed a parameter sensitivity analysis to examine if the model is robust against small perturbations and to identify parameters with critical effects on the simulations. Figure S3 shows the simulations varying the parameters up to ±10% in two scenarios (without and with 50 nM DOX). The results showed that the model was rather robust with respect to the variations in all of the parameters. This suggests that, within the explored parameter range, a critical parameter does not appear to exist in our model.

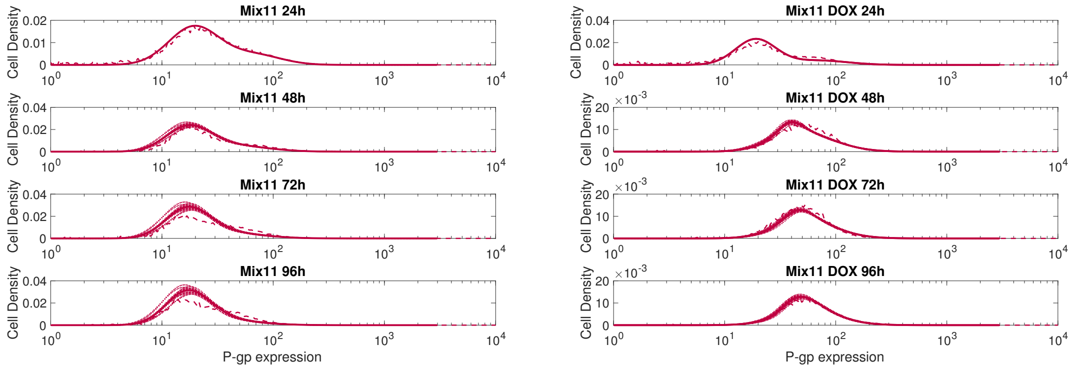

Figure S3: Sensitivity analysis for mix 1:1 cells without (left) and with (right) DOX.

Additional Results

In this section we provide additional information of the various experiments that were performed and use our model for further validations.

Experiments and simulations of assessment of cell proliferation in real-time: A least-squares method was employed to adjust our model to biological data for cell proliferation. In Fig. S4, a comparison between our model with the previously calculated parameters and experimental data is shown. In addition, in Fig. S5 it is possible to see the good agreement between our model and our *in vitro* experiments for the NCI-H460 cell line under different drug concentrations, just by adapting the therapy function. Parameters of the different simulations are collected in Table S3.

DOX	0 nM	10 nM	50 nM	100 nM
d	0	0.07	0.19	0.29
x_0	0.03	0.11	0.23	0.26

Table S3: Estimated parameter values for cell proliferation analysis, where d correspond to the value of the therapy function $\mathcal{T}(x) = d(1 - \tanh(e(x - f)))$, and $x(0) = x_0$.

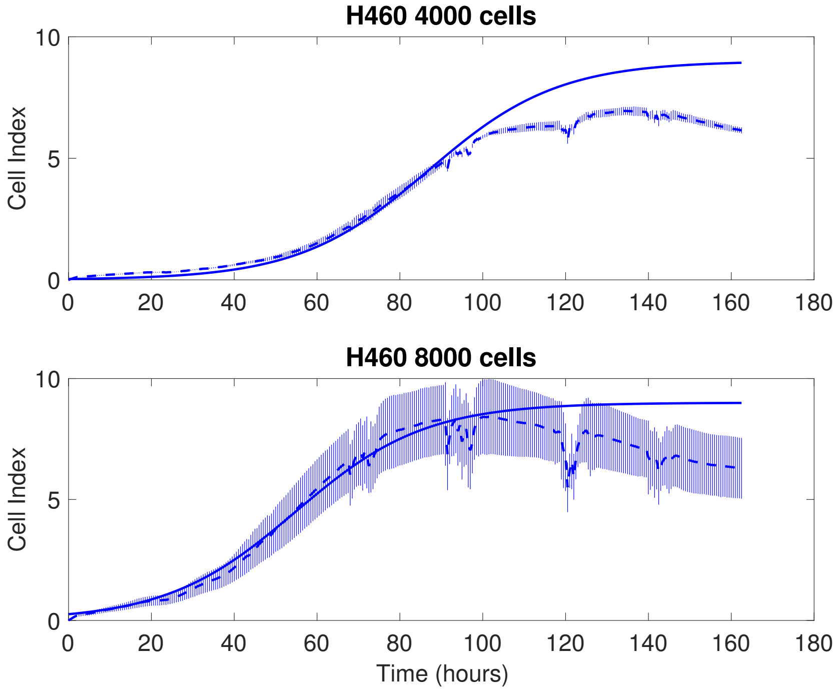

Figure S4: Fit of our model to the NCI-H460 cell line growth assessment measured by an impedance-based (xCELLigence) system with different initial cell populations. Values for the proliferation rate and saturation parameter are $\tau_1 = 15.59$ and $n_{\max} = 9$, respectively. It is important to underscore that the cell index does not exactly correspond to the true cell number (it is proportional to it within a certain dynamical range). Blue dotted and solid lines represent both the experimental data (cell index) and numerical simulation of our model, respectively. The abrupt transient drops in the experimental curves were caused by voltage fluctuations on the measuring equipment.

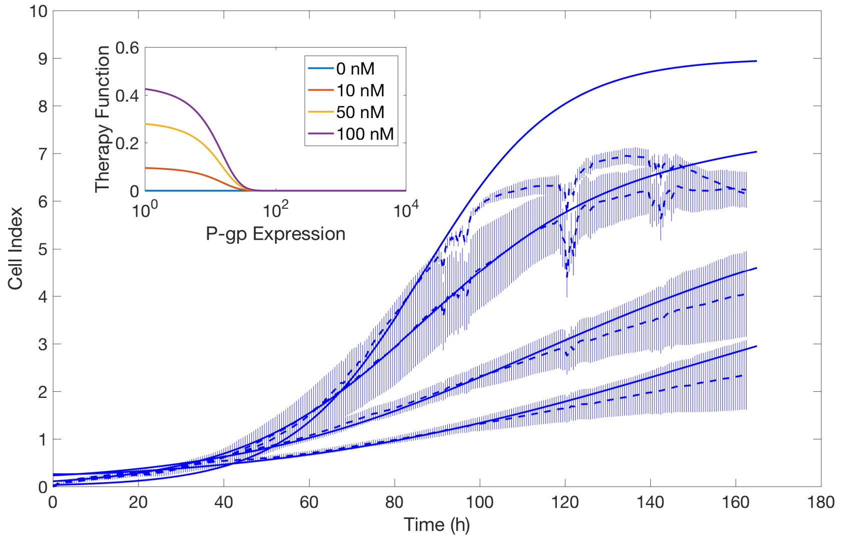

Figure S5: Fit of our model to NCI-H460 cell line growth assessment measured by a impedance-based (xCELLigence) system with 4000 initial cells and different drug conditions. Solid and dashed lines represent both the numerical simulation of our model and the experimental data, respectively. The higher the drug concentration, the lower the cell growth curve. Cell growth curves are displayed for 0 nM, 10 nM, 50 nM and 100 nM of DOX concentrations. The abrupt transient drops in the experimental curves were caused by voltage fluctuations on the measuring equipment. Inset:

Experiments and simulations of P-gp expression analyses: To model the P-gp transfer in the different cell subpopulations, its expression level under various conditions was monitored at four time points (24h, 48h, 72h and 96h). Both sensitive, resistant and mixed populations were followed in the absence/presence of drug (50 nM of DOX). In addition, to study the transfer process, the P-gp expression of the population was measured in different cell mixes (1:1, 3:1, 7:1) in the absence/presence of drug (50 nM of DOX). Figures S6-S13 below present the results and Table S4 the fitted parameters. Our model was again able to capture the tendencies of all the different scenarios.

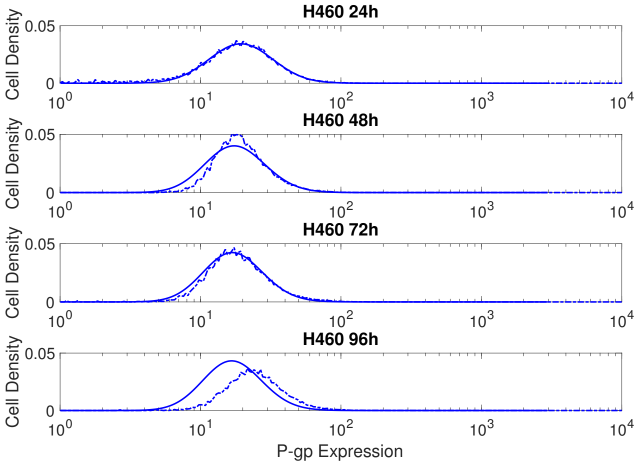

Figure S6: Evolution of sensitive cells without the drug. Dashed and solid lines represent the experimental results and the numerical simulations of our model, respectively.

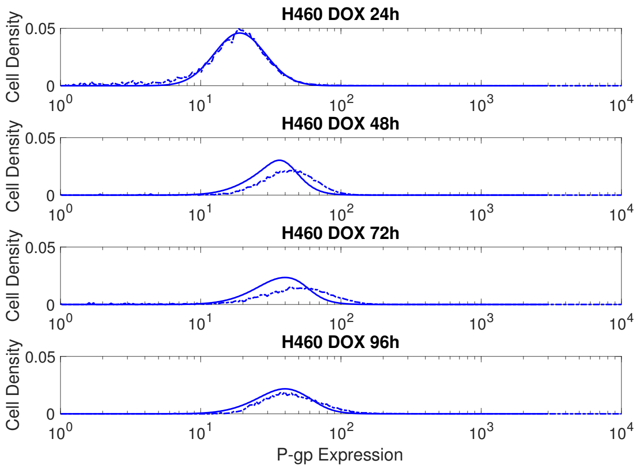

Figure S7: Evolution of sensitive cells with 50 nM DOX. Dashed and solid lines represent the experimental results and the numerical simulations of our model, respectively.

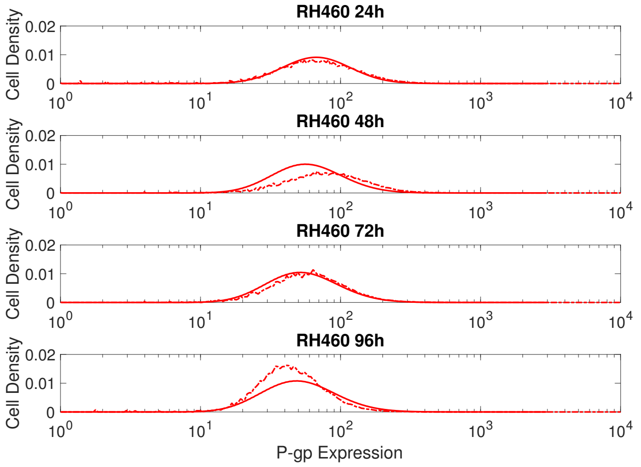

Figure S8: Evolution of resistant cells without the drug. Dashed and solid lines represent the experimental results and the numerical simulations of our model, respectively.

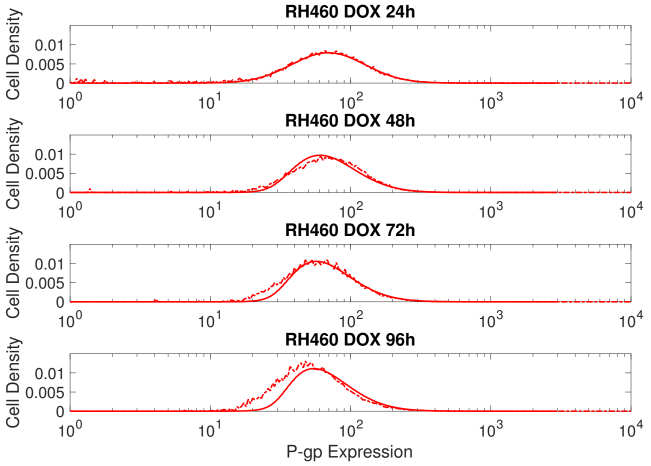

Figure S9: Evolution of resistant cells with 50 nM DOX. Dashed and solid lines represent the experimental results and the numerical simulations of our model, respectively.

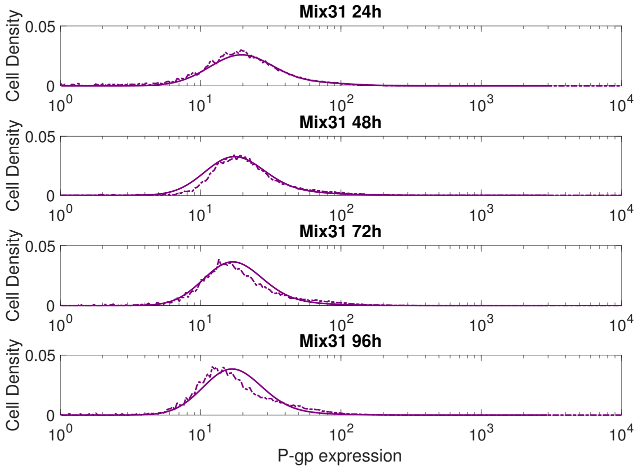

Figure S10: Evolution of mix 3:1 cells without drug. Dashed and solid lines represent the experimental results and the numerical simulations of our model, respectively.

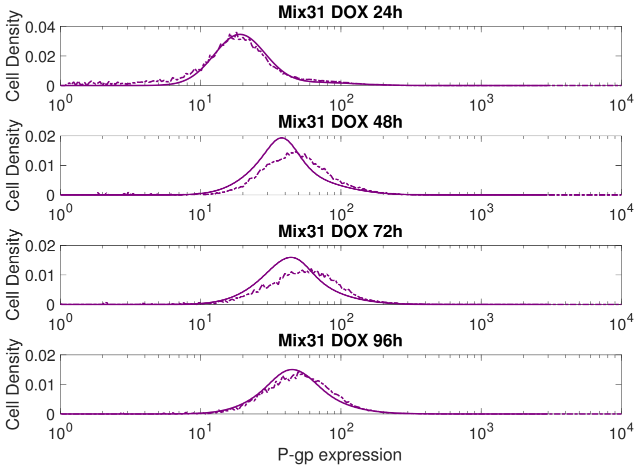

Figure S11: Evolution of mix 3:1 cells with 50 nM DOX. Dashed and solid lines represent the experimental results and the numerical simulations of our model, respectively.

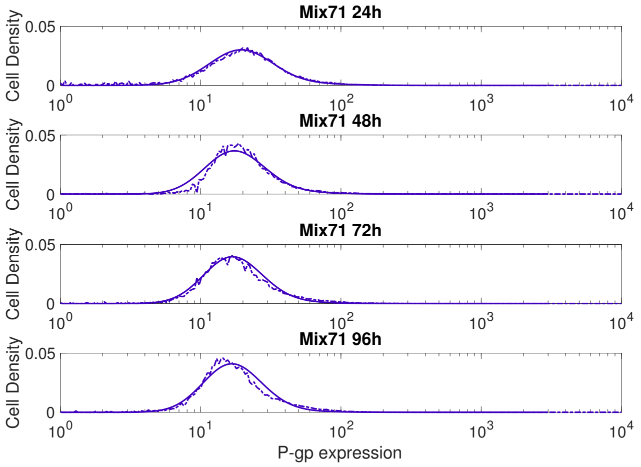

Figure S12: Evolution of mix 7:1 cells without drug. Dashed and solid lines represent the experimental results and the numerical simulations of our model, respectively.

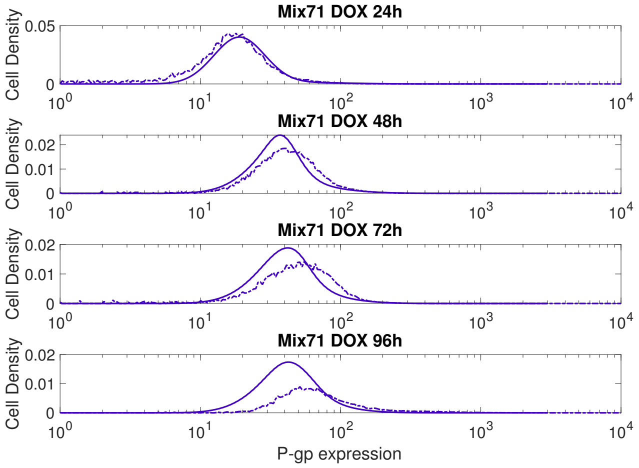

Figure S13: Evolution of mix 7:1 cells with 50 nM DOX. Dashed and solid lines represent the experimental results and the numerical simulations of our model, respectively.

**Parameter**	**Description**	**Sensitive**	**Resistant**
Z Basal	P-gp distribution mean basal value	20	95.52
σ	P-gp distribution standard deviation basal value	0.35	0.5
τ	Cell doubling time	22.27	35.09
δ	P-gp splitting factor	2	2
	**Induction function** $v_I(x) = a(1 - \tanh(b(x - c)))$		
a	Induction function parameter	0.5	0.5
b	Induction function parameter	0.01	0.01
c	Induction function parameter	240	10
	**Therapy function** $\mathcal{T}(x) = d(1 - \tanh(e(x - f)))$		
d	Therapy function parameter	0.45	0.45
e	Therapy function parameter	0.07	0.07
f	Therapy function parameter	8	8
	**Transfer function** $v_{T_i}(x) = -g_d(\tanh(h(x - x_0))) + g_d(2 - i)$		
$g_{d=0\,\mathrm{nM}}$	Transfer function parameter	0.01	2.5
$g_{d=50\,\mathrm{nM}}$	Transfer function parameter	0.1	2.5
h	Transfer function parameter	3	0.01
x_0	Transfer function parameter	90	5

Table S4: Estimated parameters' values for P-gp expression analysis

Experiments with medium exchange: In these experiments it was possible to observe how the MVs shed by resistant cells affect the P-gp expression in different populations. Resistant cells were seeded in a culture medium during 24h and removed afterwards. In such a conditioned medium sensitive cells or different mixes were placed to check their behaviour, in the absence or in the presence of the drug (DOX). It was apparent that in the presence of DOX, sensitive cells were more likely to internalize MVs from the microenvironment. Figure 5 in the main text displays the effect in the sensitive population and the transfer functions used to fit the data. The values of the different parameters employed in the simulation are collected in Table S5.

Parameter	Description	Value
Z Basal	P-gp distribution mean basal value	3.38
σ	P-gp distribution standard deviation basal value	0.41
	**Induction function** $v_I(x) = a(1 - \tanh(b(x-c)))$	
a	Induction function parameter	0.08
b	Induction function parameter	0.01
c	Induction function parameter	5
	**Therapy function** $\mathcal{T}(x) = d(1 - \tanh(e(x-f)))$	
d	Therapy function parameter	0.08
e	Therapy function parameter	0.07
f	Therapy function parameter	5
	**Transfer function** $v_{T_i}(x) = -g_d(\tanh(h(x-x_0))) + g_d(2-i)$	
$g_{d=0\,\text{nM}}$	Transfer function parameter	0.01
$g_{d=50\,\text{nM}}$	Transfer function parameter	0.1
h	Transfer function parameter	0.01
x_0	Transfer function parameter	50

Table S5: Estimated parameters values for medium exchange analysis

To test for reproducibility, three additional independent replicas were carried out (besides the one depicted in Fig. 5). The results showed very similar behaviour in all cases and identical conclusions were obtained in the statistical analyses. Figure S14 collects the comparisons of the four experimental runs (left column) together with the corresponding confidence intervals and the found statistically significant differences.

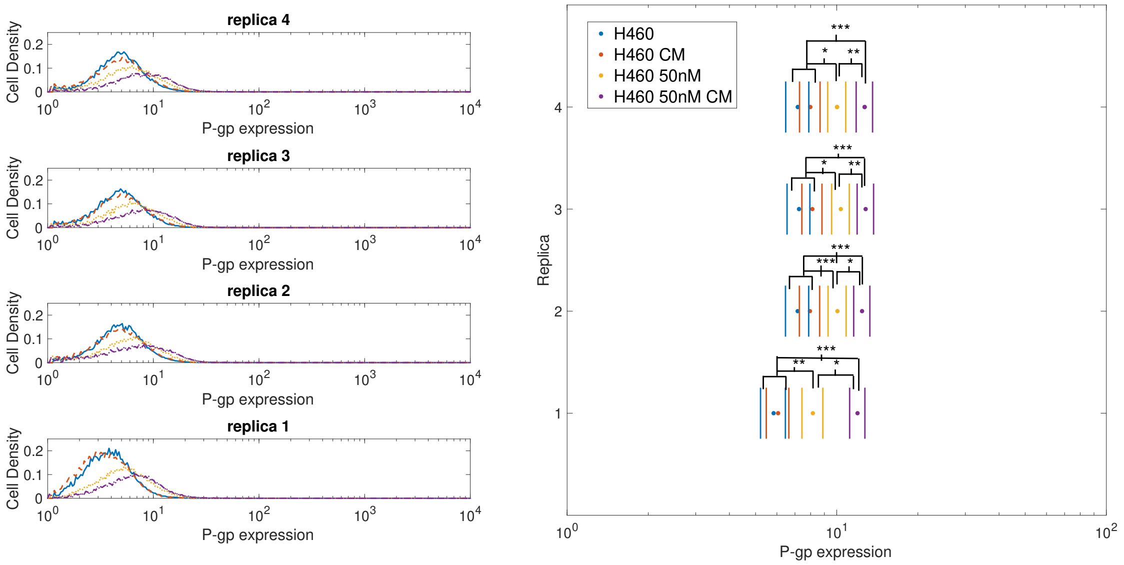

Figure S14: Detection of P-gp transfer during 48 h in different culture media with sensitive cells ($\simeq 2 \times 10^4$) in four independent replicas. The left column represents the P-gp distributions in four considered scenarios: only the H460 cells (blue solid curves), H460 cells grown in a conditioned medium (CM) exchanged from the H460/R cells to the H460 cells medium (red dashed curves), H460 cells grown in the presence of 50 nM of DOX (yellow dotted curves) and H460 cells grown both in the presence of 50 nM of DOX and CM (violet dashed-dotted curves). The right column collects the confidence intervals (at the confidence level of 95%) in the four replicas and the four considered scenarios. The asterisks denote the p-values for pairwise comparisons: $p < 0.05$ (*), $p < 0.005$ (**) and $p < 0.0005$ (***).

Differential labelling of sensitive and resistant cells: A CellTrace^{TM} Violet reagent was used to differentially label NCI-H460 sensitive cells, when grown isolated and in mixture 1:1 with resistant cells, in the absence/presence of DOX. The aim here was to observe (data not shown) changes in the profiles of sensitive cells, as shifts to lower values of the CellTrace^{TM}, reflecting the fact that the label content per cell decreased due to proliferation. We observed that in the presence of DOX, sensitive cells displayed smaller shifts when compared with those in the absence of DOX. This evidenced a selection pressure which was higher in those cells having a higher proliferation rate. Since resistant cells were not labelled, the obtained profiles did not change either in the absence/presence of DOX. When mixing sensitive and resistant cells, a similar shift towards lower values of the CellTrace^{TM} was observed for sensitive cells in the absence of DOX. In the presence of DOX, not only a smaller shift was apparent, but also the relative importance of the sensitive cells with respect to the resistant cells was quite noticeable. This experiment provided further validation of our mathematical model.

Response to different treatment protocols: In these simulations we observed the response to three different DOX protocols under varying initial configurations of sensitive and resistant cells. Drug administration in these protocols is depicted in Fig. S15.

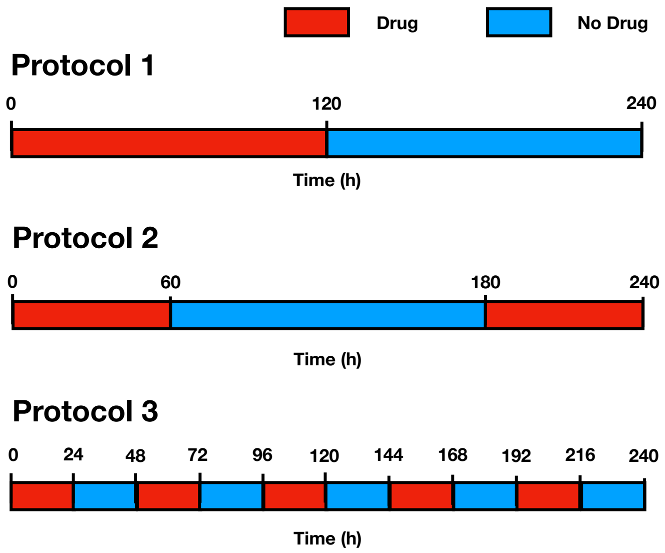

Figure S15: Drug administration scheduling in the three protocols considered.

In Fig. S16 it is possible to see how the three considered protocols can result in very different responses to treatment, even though the total time under drug stress, as well as the total dose, is the same in all of them. The biggest differences were found in the simulations with only sensitive cells and high DOX concentration, and the smallest in resistant cells with low DOX concentration. Cell mixes yielded intermediate evolving scenarios.

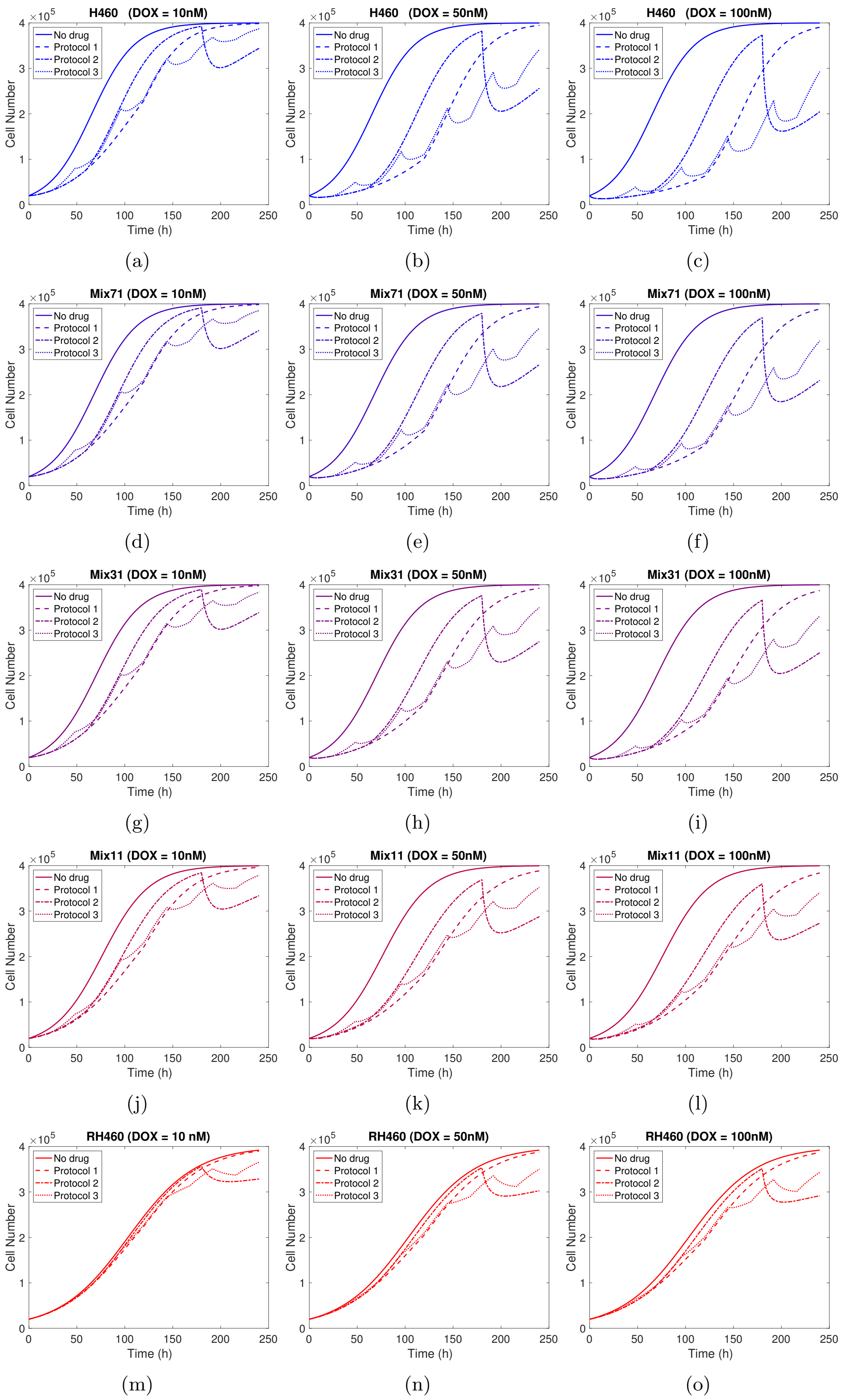

Figure S16: Treatment response for the control (no drug) and the three considered protocols for H460 (first row), Mix 7:1 (second row), Mix 3:1 (third row), Mix 1:1 (fourth row) and RH460 (fifth row) initial populations when administering three different concentrations of DOX. The initial cell number was 20000 cells in all cases.

To further quantify the relative importance of the role played by non-genetic processes on the total cell number, additional simulations were carried out. To this end, Lamarkian induction and MV transfer processes were independently switched off (see Figs. S17 and S18, respectively) in our model equations (i.e. $v_{I_i} = 0$ and $v_{T_i} = 0$, respectively), and the differences in the cell number were compared with the scenario were both processes were present (always in combination with Darwinian selection, which was never switched off).

The effect of induction on cell number was, in all the examined cases, 3-6 times larger than MV transfer in the considered time window (240 h). Also, these processes displayed a higher impact on those populations containing a larger fraction of sensitive cells (i.e., in H460 and Mix7:1). When analyzing the three different protocols, we observed that protocols 1 and 3 showed a similar behaviour if Lamarkian induction was suppressed (see Fig. S17), whereas protocols 1 and 2 performed comparably if MV transfer was absent (see Fig. S18).

Moreover, the effect of the three administered doses (10, 50 and 100 nM of DOX) on the relevance of Lamarkian induction and MV transfer processes is interesting. As the doses increased, the relative importance of induction in Mix3:1 and Mix1:1 decreased (thus displaying and inverse relation, see Fig. S17), whereas the relevance of MV transfer only decreased noticeably in Mix1:1 (Fig. S18). However, in the rest of the populations the larger differences were reached with a concentration of 50 nM. This suggests the existence of an optimal dose that would minimize the effect of non-genetic transformations when the relative fraction of sensitive cells is large. In contrast, when resistant cells constitute the majority of the total tumor cell population, these processes are always minimized with the higher dose possible.

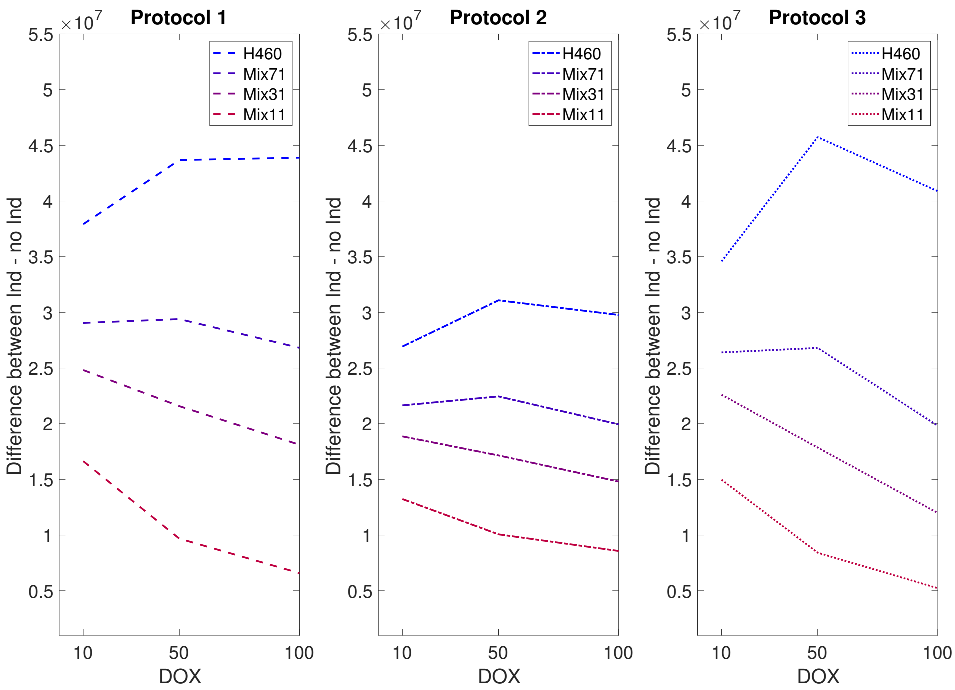

Figure S17: Accumulated deviation in cell number for a period of 240 h when all MDR processes are present versus the case when Lamarkian induction is absent. The curves have been calculated using the L_2 norm of the difference between the curves shown in Fig. S16 with those in which induction has been inactivated. All parameters correspond to Fig. S16.

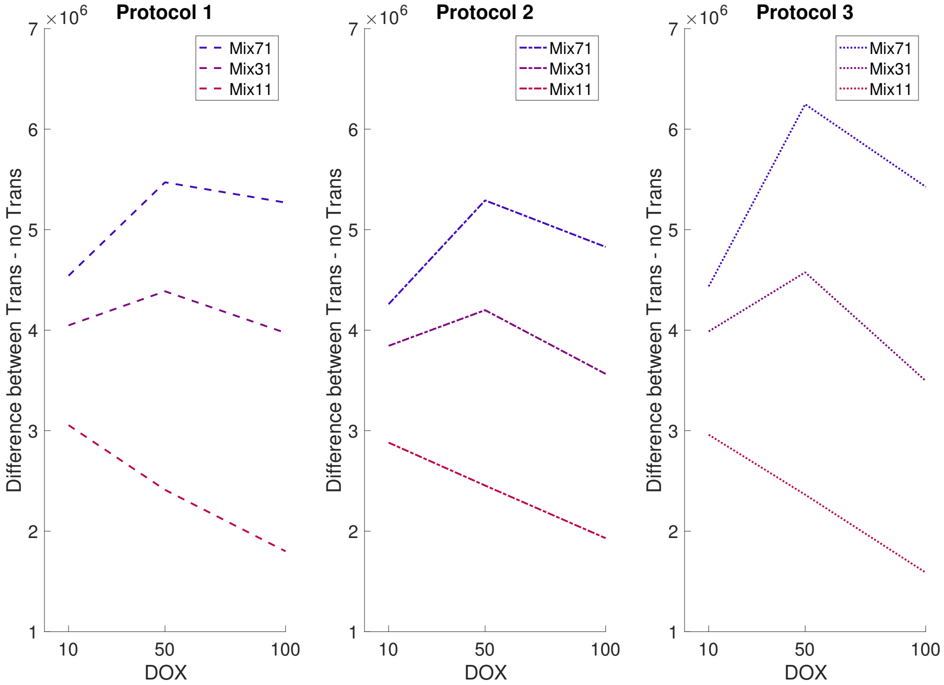

Figure S18: Accumulated deviation in cell number for a period of 240 h when all MDR processes are present versus the case when MV transfer is absent. The curves have been calculated using the L_2 norm of the difference between the curves shown in Fig. S16 with those in which transfer has been inactivated. All parameters correspond to Fig. S16.

Bibliography

[Bellomo *et al.*, 2008] N. Bellomo, N.K. Li, P.K. Maini. (2008) On the foundations of cancer modelling: Selected topics, speculations, and perspectives, *Mathematical Models and Methods in Applied Sciences*, **18**, 593-646.

[Lavi *et al.*, 2013] O. Lavi, J.M. Greene, D. Levy, M. Gottesman. (2013) The role of cell density and intratumoral heterogeneity in multidrug resistance, *Cancer Research*, **73**, 7168-7175.

[Chisholm *et al.*, 2015] R.H. Chisholm, T. Lorenzi, A. Lorz, A.K. Larsen, L.N. de Almeida, A. Escargueil, J. Clairambault. (2015) Emergence of drug tolerance in cancer cell populations: An evolutionary outcome of selection, nongenetic instability, and stress-induced adaptation, *Cancer Research*, **75**, 930-939.
